# Ferroelectricity and Piezoelectric Energy Harvesting of Hybrid A_2_BX_4_-Type Halogenocuprates Stabilized by Phosphonium Cations

**DOI:** 10.1021/acsmaterialsau.1c00046

**Published:** 2021-11-08

**Authors:** Supriya Sahoo, Thangavel Vijayakanth, Premkumar Kothavade, Prashant Dixit, Jan K. Zaręba, Kadhiravan Shanmuganathan, Ramamoorthy Boomishankar

**Affiliations:** ^†^Department of Chemistry and ^‡^Centre for Energy Science, Indian Institute of Science Education and Research (IISER), Dr. Homi Bhabha Road, Pune 411008, India; § Polymer Science and Engineering Division, CSIR-National Chemical Laboratory, Dr. Homi Bhabha Road, Pune 411008, India; ∥ Academy of Scientific and Innovative Research, Ghaziabad 201002, India; ⊥ PZT Centre, Armament Research and Development Establishment, Dr. Homi Bhabha Road, Pune 411021, India; # Advanced Materials Engineering and Modelling Group, Wrocław University of Science and Technology, Wybrzeże Wyspiańskiego 27, 50-370 Wrocław, Poland

**Keywords:** Hybrid Perovskite, Phosphonium Salt, Ferroelectric, Piezoelectric, Energy Harvesting

## Abstract

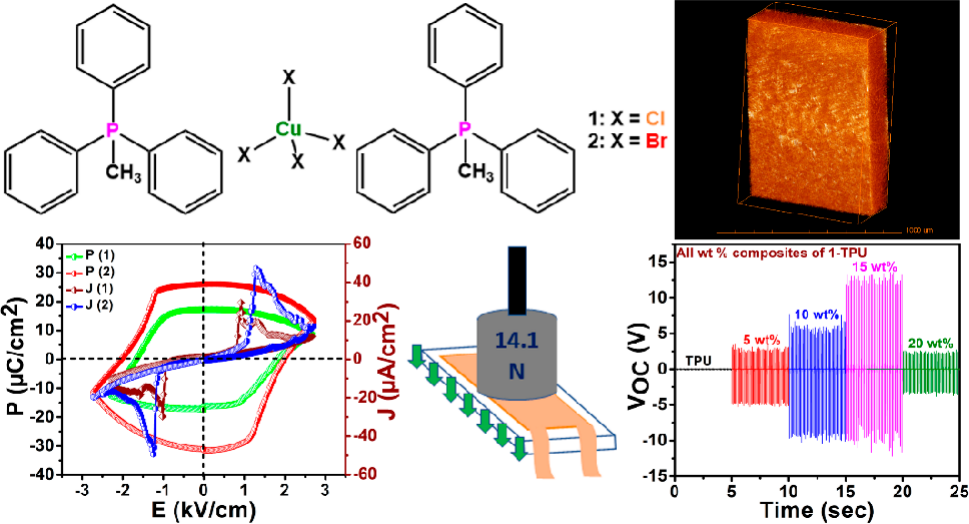

Perovskite-structured compounds containing organic cations and inorganic anions have gained prominence as materials for next-generation electronic and energy devices. Hybrid materials possessing ferro- and piezoelectric properties are in recent focus for mechanical energy harvesting (nanogenerator) applications. Here, we report the ferroelectric behavior of A_2_BX_4_-type halogenocuprate materials supported by heteroleptic phosphonium cations. These lead-free discrete Cu(II) halides [Ph_3_MeP]_2_[CuCl_4_] (**1**) and [Ph_3_MeP]_2_[CuBr_4_] (**2**) exhibit a remnant polarization (*P*_r_) of 17.16 and 26.02 μC cm^–2^, respectively, at room temperature. Furthermore, flexible polymer films were prepared with various weight percentage (wt %) compositions of **1** in thermoplastic polyurethane (TPU) and studied for mechanical energy harvesting applications. A highest peak-to-peak voltage output of 25 V and power density of 14.1 μW cm^–2^ were obtained for the optimal 15 wt % **1**-TPU composite film. The obtained output voltages were utilized for charging a 100 μF electrolytic capacitor that reaches its maximum charging point within 30 s with sizable stored energies and accumulated charges.

## Introduction

Materials exhibiting photovoltaic, thermoelectric, electrochemical, and piezoelectric properties represent a burgeoning area of research due to their ability to harvest various forms of energy via the conversion of their optical, thermal, chemical, and mechanical effects into electrical outputs.^1−4^ Recent activity in this area is largely focused on the development of hybrid perovskites since they combine the advantages of both the organic and inorganic substances, yielding structurally flexible materials.^5−9^ They also offer solution and low-temperature processability, strong optical absorption, high carrier mobility, and tunable optical properties and band gap.^3,10−15^ Particularly, devices fabricated from hybrid emissive materials have led to remarkable developments in perovskite solar cells with high power conversion efficiencies.^11,16−19^ Lately, these classes of materials are explored for harvesting energies from other forms of forces such as thermoelectric and piezoelectric effects.^2,15,17,20,21^

Piezoelectricity is an efficient source of alternate energy, wherein the electrical energy is produced via the application of external stress, including natural forces and biomechanical movements.^22−24^ In the past, nanogenerators of this type were prepared using traditional piezoelectric and ferroelectric ceramic materials like barium titanate, lithium niobate, lead titanate, lead zirconate titanate, and zinc oxide.^25−28^ However, the presence of heavy weight or toxic elements and the poor nanogenerator attributes for some of these substances have triggered the search for alternative class of materials.^11,29^ Even though the use of organic polymers such as polyvinylidene fluoride (PVDF) and its copolymers has resulted in notable device characteristics, these materials require additional stimuli such as mechanical stretching, high-temperature annealing, and external additives to obtain the piezoelectrically active β-phase.^30−36^ Hence, organic–inorganic hybrid materials with piezoelectric properties serve as an attractive platform to fabricate devices for these applications.^5,6,37^

Hitherto, several nanogenerator devices have been fabricated using lead- and tin-based hybrid materials.^38−41^ For example, a composite device based on MASnI_3_ embedded in the PVDF was shown to yield an output peak-to-peak voltage of 12 V and a power density of 21.6 μW cm^–2^.^42^ However, the toxic nature of lead and the intrinsic instability of Sn(II)-halides have limited the use of such perovskites for practical energy harvesting applications.^4,24,32^ Alternatively, hybrid systems derived from first-row metal ions could offer an attractive protocol to concomitantly mitigate the environmental and stability concerns.^43−45^ Our lab recently designed an A_4_MX_6_-type ferroelectric phosphonium pseudohalogenometallate salt [Ph_3_PMe]_4_[Ni(NCS)_6_], which shows a ferroelectric polarization of 18.71 μC cm^–2^ and a maximum nanogenerator output voltage of 19.29 V for its polymer composite with thermoplastic polyurethane (TPU).^46^ The high output voltage of this composite is attributed to its ferroelectric nature as the piezoelectric coefficient is proportional to the remnant polarization of a material. Inspired by this discovery, we set out to explore novel ferroelectric halogeno- and pseudohalogenometallate compounds derived from 3d-metal ions for mechanical energy harvesting applications. Although several examples of the discrete and polymeric halogenometallates are reported, more often than not the solution processing of these compounds for device fabrication is complicated by the reactivity of the metal–halogen bonds.^28,47^

Herein, we report the ferroelectric behavior of two discrete halogenocuprates, [Ph_3_PMe]_4_[CuCl_4_] (**1**) and [Ph_3_PMe]_4_[CuBr_4_] (**2**), supported by *C*_3_ symmetric methyltriphenyl phosphonium cations. The polarization versus electric field (*P–E*) hysteresis loop measurements on these compounds gave the remnant polarization values of 17.16 and 26.02 μC cm^–2^ for **1** and **2**, respectively, at room temperature. Furthermore, polymer composites with various weight percentages of **1** were prepared with TPU and studied for mechanical energy harvesting applications. These studies gave the highest open-circuit peak-to-peak voltage of 25 V for the 15 wt % polymer composite film of **1**. These polymer composites were found to give pronounced current density and power density values, as well. Finally, the output voltage generated from the 15 wt % **1**-TPU device was utilized to charge a 100 μF capacitor after rectification, which gave stored energy of 162 μJ and the measured charge of 180 μC. To the best of our knowledge, halogenocuprate salts templated by phosphonium cations presented here display the highest nanogenerator output performance among first-row transition metal ion containing hybrid halogenometallates.^48^

## Experimental Section

### General Remarks

Methyltriphenylphosphonium chloride and methyltriphenylphosphonium bromide were purchased from Sigma-Aldrich and were used without further purification. The other starting materials such as copper(II) chloride and copper(II) bromide were purchased from Avra Chemicals. TPU was purchased from BASF and was used as received. The NMR data for the phosphonium compounds were recorded on a Bruker 400 or JEOL 400 MHz spectrometer (^31^P{^1^H} NMR using 85% H_3_PO_4_ (^31^P) as internal standards). The thermogravimetric analyses (TGA) were performed using the PerkinElmer STA-6000 analyzer at a heating rate of 10 °C/min in a nitrogen atmosphere. Melting point analyses were done using a Buchi M-560 melting point apparatus and were uncorrected. The variable-temperature powder X-ray diffraction (VT-PXRD) data were measured in the 2θ range of 5 to 50° on a Bruker-D8 Advance X-ray diffractometer. The field-emission scanning electron microscopy (FE-SEM) analysis of all the piezo- and ferroelectric crystallites and their composite films (all different wt %) was performed using the Zeiss ultra plus FE-SEM instrument with a minimum spatial resolution of 1 μm. The 3D X-ray microtomography analyses were performed using a Carl Zeiss Versa 510 microscope with an applied X-ray energy of 80 kV. The static mechanical testing (stress–strain behavior) of pure TPU and the polymeric composite films was performed on an Instron 5943 model universal testing machine using rectangular film strips (0.2 mm thickness, 5 mm width and 10 mm gauge length) at a 20 mm/min strain rate.

### General Synthetic Procedure for the Preparation of Phosphonium Salts with Tetrahalo Copper(II) Anions

The tetrahalo copper(II) salts of methyltriphenylphosphonium cation were prepared as per the reported procedure.^49^ Single crystals of **1** and **2** were obtained by slowly evaporating an ethanolic solution containing stoichiometric amounts (2:1) of methyltriphenylphosphonium chloride and methyltriphenylphosphonium bromide with copper chloride and copper bromide, respectively. For **1** and **2**, yellow and violet-colored crystals suitable for single-crystal X-ray diffraction were obtained after 7 days. All of the details about the two phosphonium salts, including the analytical data, can be found in the Supporting Information.

### Crystallography

The single-crystal X-ray diffraction data for **1** and **2** at 100 K were obtained on a Bruker Smart Apex Duo diffractometer using Mo Kα radiation (λ = 0.71073 Å). Crystal structures were solved using the direct method and then refined by full-matrix least-squares against *F*^2^ using SHELXL-2014/7 built in the Apex 3 program.^50^ All of the non-hydrogen atoms were refined anisotropically. Hydrogen atoms were fixed in geometric positions to their parent atoms using riding model.^51^ Crystals of **1** diffracted weakly at higher angles, and hence the data were truncated to 2θ = 50°. The structural illustrations were prepared using DIAMOND-3.1 software.

### General Procedure for the Preparation of Polymer Composite Films and Devices

The preparation of the composite films of **1**-TPU involves the dissolution of appropriate quantities of the ferro- and/or piezoelectric crystallites into a solution containing TPU in dimethylformamide (DMF). Mechanical stirring of this mixture at 70 °C for 15 min, followed by vortex mixing for 15 min, results in a homogeneous solution. The solutions were then poured onto a Petri dish and kept undisturbed in an oven at 70 °C for 5 h. The dried free-standing composite films comprising 5, 10, 15, 20, and 25 wt % of **1** in TPU were subsequently peeled off from the glass Petri dish. To complete the device architecture, copper adhesive tapes were placed on either side of the composite films along with the electrical contacts. Finally, the electrodes were covered with a 2 mm thick polydimethylsiloxane (PDMS) polymer. For comparison, a device made up of a neat TPU polymer film encapsulated with PDMS was also prepared and examined under identical experimental conditions.

### Nonlinear Optical Measurements

Nonlinear optical studies were performed using an attenuated output from a Coherent Astrella Ti:sapphire regenerative amplifier providing 800 nm laser pulses at 1 kHz repetition rate and of 75 fs duration.

A Kurtz-Perry test was performed at 298 K. Potassium dihydrogen phosphate (KDP) was used as a second harmonic generation (SHG) reference. The single crystals of **1** and **2** and those of KDP were crushed with a spatula and sieved through a mini-sieve set (Aldrich), collecting a microcrystal size fraction of 250–177 μm. Next, size-graded samples were fixed between microscope glass slides (forming tightly packed layers), sealed, and mounted to the sample holder. An average power of an 800 nm beam, equal to 245 mW, - with a spot area of 0.5 cm^2^ was used for the Kurtz-Perry study. The laser beam was directed onto samples at 45° and was unfocused in all cases. Signal-collecting optics, mounted to the glass optical fiber, were placed perpendicularly to the plane of the sample (backscattering geometry), which was placed on a horizontally aligned holder. Scattered pumping radiation was suppressed with the use of a 750 nm short-pass dielectric filter (FESH0750, Thorlabs). The emission spectra were recorded by an Ocean Optics Flame T spectrograph.

### Ferroelectric, Dielectric, and Piezoelectric Measurements

The powder samples of **1** and **2** were compacted in the form of discs (of approximately 8 mm diameter and 1.2 mm thickness) and electroded with Al adhesive tapes. The ferroelectric polarization versus electric field (*P–E*) and fatigue measurements were performed on a aixACCT TF-2000E model hysteresis loop analyzer. The leakage currents were dynamically recorded during the hysteresis loop measurements.

The dielectric permittivity measurements were performed on the pressed powder pellets of **1** and **2**. The measurements were performed using the Solartron analytical impedance analyzer 1260 coupled with a dielectric interface 1296A operating with the Janis 129610A cryostat sample holder and a Lakeshore 336 model temperature controller.

The *d*_33_ measurements were performed on a Piezotest meter model PM300 on the compacted discs (of approximately 8 mm diameter and 1.2 mm thickness) of **1** and **2**. The piezoelectric energy harvesting measurements were conducted on a custom-built periodic impact instrument. The output voltages and currents were measured using a Tektronix 2024 mixed signal oscilloscope operating at an input impedance of 1 MΩ. The thickness and the active area of the devices under test were ∼1 mm and 360 mm^2^, respectively.

The presence of polarization on **1** and **2** was confirmed from the preliminary theoretical dipole moment ONIOM calculations performed using the Gaussian 09 program. In this process of calculation, the anionic parts were taken as the high layer and the cationic parts were regarded as the lower layer. Dipole moment (ONIOM) calculations were performed using density functional theory (DFT) methods.^52^

## Results and Discussion

### Syntheses, Characterization, Crystal Structures, and Hirshfeld Surface Analyses

**Figure 1:**
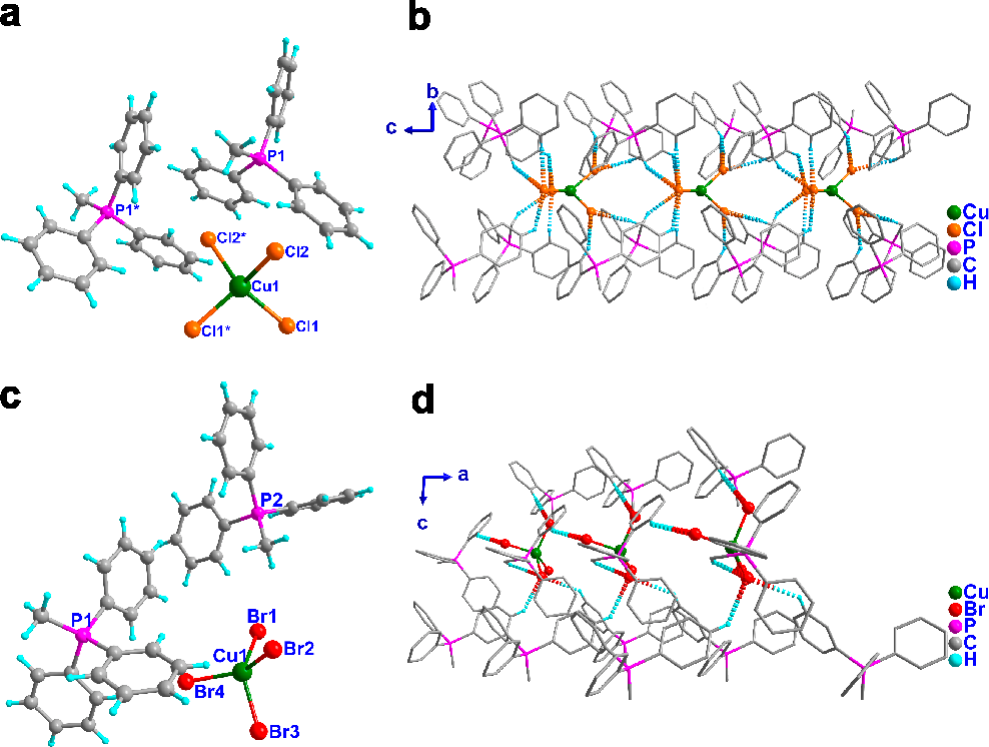
Molecular structures of (a) **1** and (c) **2** at 100 K. The hydrogen bonding interactions in (b) **1** and (d) **2** mediated by C–H···X interactions (**1**: X = Cl and **2**: X = Br).

The phosphonium salts **1** and **2** were obtained as yellow- and violet-colored crystals, respectively, from the corresponding phosphonium- and metal-halide precursors by following the procedure reported previously (Scheme S1).^49,53^ The purity of the synthesized materials was determined from ^31^P NMR spectroscopy (Figures S1 and S2), and powder X-ray diffraction analyses. The room-temperature (298 K) structures of **1** and **2** were reported in polar orthorhombic *Fdd*2 and monoclinic *Cc* space groups, respectively.^15^ The molecules consist of two phosphonium cations and noncoordinating (CuCl_4_)^2–^ and (CuBr_4_)^2–^ ions for **1** and **2**, respectively (Figure 1a,c). A closer inspection of the structure of **1** and **2** at 298 K indicated that the presence of lower-symmetric heteroleptic phosphonium cations, and the distorted tetrahedral geometries at the [CuX_4_]^2–^ centers have contributed to the overall acentricity of their crystal systems.

To track the existence of any centrosymmetric phase in these structures, we performed the variable-temperature single-crystal X-ray diffraction (VT-SCXRD) analyses on the crystals of **1** and **2** between the temperatures 100 and 400 K (Figures S6 and S7). These analyses confirmed no space group change for both **1** and **2** at all of the measured temperatures, albeit with a marginal increase in their cell volumes and some unit cell axes (Figures S6 and S7). Such changes can be attributed to the thermal motion of atoms at higher temperatures that causes volume expansions in the unit cells. In a similar manner, the variable-temperature powder X-ray diffraction (VT-PXRD) profile of **1** and **2** did not exhibit any phase change or systematic absence of peaks upon increasing the temperatures from 296 to 423 K and 296 to 383 K, respectively (Figures S8a and S9a). The TGA of **1** and **2** reveals that these compounds are stable for temperatures up to 523 and 550 K, respectively, and exhibit no additional heat anomalies (Figures S8b and S9b).

A closer inspection of the 100 K structures of **1** and **2** revealed the presence of strong nonclassical C–H···X (X = Cl or Br) interactions (Figure 1b,d). The structure of **1** shows a 1D-hydrogen-bonded assembly along the *c*-axis. Each CuCl_4_ unit interacts with eight phosphonium units via C–H···Cl interactions in such a way that the anionic chlorocuprates are entirely encapsulated by a hydrophobic network of phosphonium cations. The crystal structure of **2** also exhibits a 1D-hydrogen-bonded structure mediated by C–H···Br interactions along the *b*-axis. However, each CuBr_4_ unit in it is associated with five phosphonium moieties.

**Figure 2:**
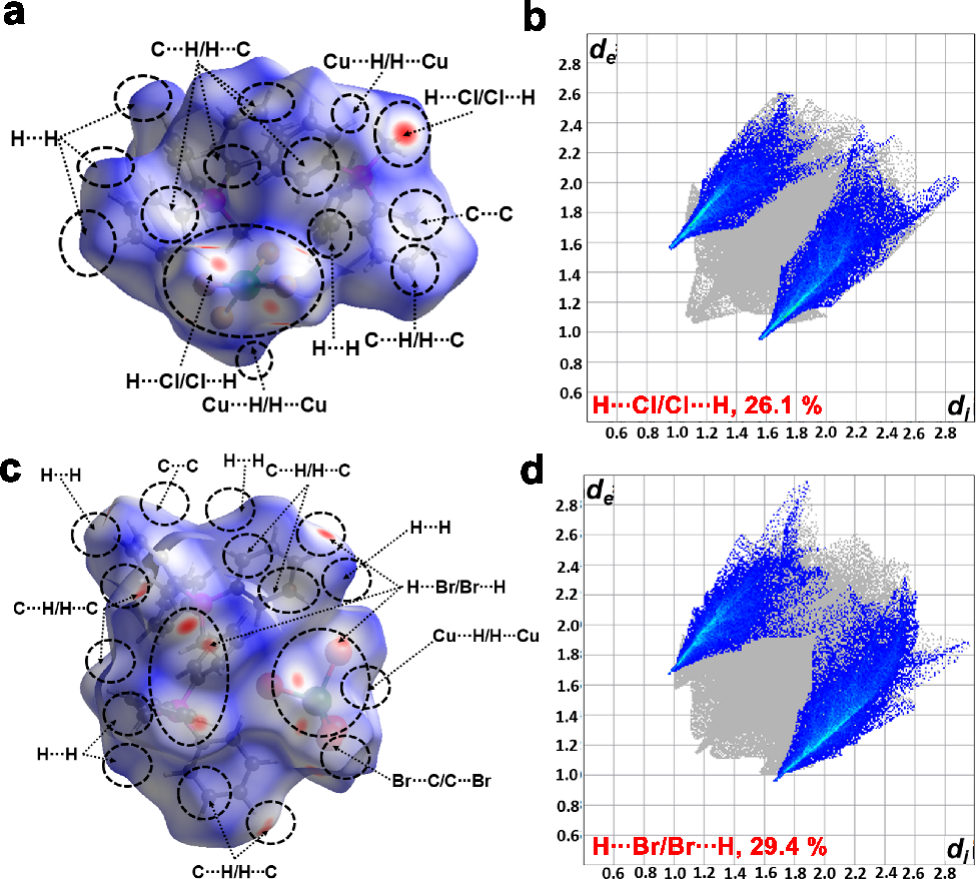
(a,c) Hirshfeld surface views and (b,d) 2D fingerprint plots of C–H···Cl and C–H···Br interactions in **1** and **2**, respectively.

Furthermore, the quantification of various kinds of molecular interactions present in **1** and **2** were visualized through *d*_norm_-mapped Hirshfeld surfaces and by analyzing the 2D fingerprint plots using the Crystal Explorer 3.1 program. The C–H···Cl and C–H···Br intermolecular hydrogen bonds are indicated by the bright red colored spots on the *d*_norm_-mapped Hirshfeld surface for **1** and **2**, respectively (Figure 2a,c). These interactions include C···C, C···H, H···H, Cu···H/H···Cu, H···Cl/Cl···H (2.1, 23.7, 46.7, 1.3, 26.1%) and C···C, C···H, H···H, Cu···H/H···Cu, C···Br/Br···C,H···Br/Br···H (1.1, 22.9, 44.6, 1.1, 0.9, 29.4%) for **1** and **2**, respectively (Figures S11, S12, S14, and S15). Among all of the interactions, the H···Cl interactions in **1** contribute 26.1% and the H···Br interactions in **2** contribute 29.4% of the overall interactions (Figure 2b,d). The dispersion and van der Waals contacts account for the remaining interactions. Notably, the hydrogen bonding (C–H···Cl and C–H···Br) and ionic interactions account for strong long-range order with structure stabilization energy of 3–9 kJ/mol.^54^ Though it appears that the dispersion and van der Waals contacts contribute to the majority of the interactions present in **1** and **2**, they account for short-range order in the molecules with lower stabilization energy of 0.4–4 kJ/mol.

### Second Harmonic Generation, Ferroelectric, Dielectric, and Piezoelectric Studies

To further validate the acentric structure of both **1** and **2**, second harmonic generation (SHG) measurements were performed at room temperature using a Kurtz-Perry-type measurement.^55^ The size-graded powders of **1** and **2** gave the respective SHG efficiencies of 5.1 × 10^–3^ and 0.028 with respect to standard KDP sample upon irradiation with 800 nm, 1 kHz laser with a pulse width of 75 fs. The observation of the SHG signals at room temperature serves as strong confirmation of the noncentrosymmetric structures of both of these solids (Figure S16).

**Figure 3:**
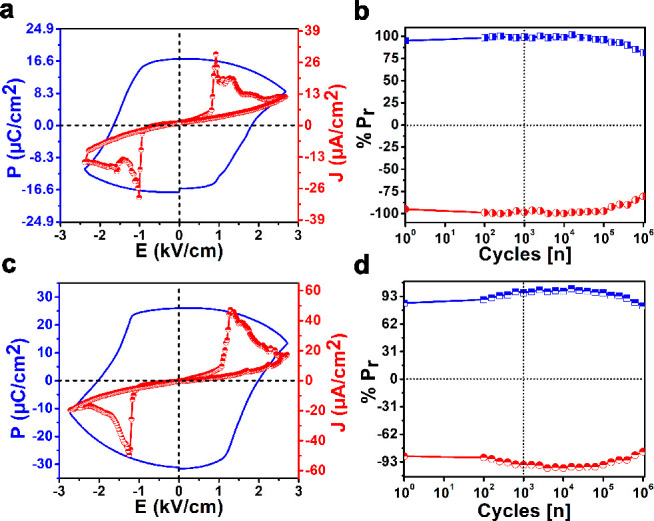
*P–E* hysteresis loop measurements and leakage current densities of (a) **1** and (c) **2** at room temperature. Ferroelectric fatigue measurements of (b) **1** and (d) **2** for up to 10^6^ cycles.

The point group symmetries of **1** and **2** were *C*_2*v*_ and *C*_*s*_, respectively, which are one of the 10 polar point groups suitable for ferroelectric studies.^56^ The *P*–*E* hysteresis loop measurements were performed on their powder pressed pellets using a Sawyer-Tower circuit setup. These measurements gave a rectangular hysteresis loop for both **1** and **2** at room temperature. The remnant polarization (*P*_r_) values of 17.16 and 26.02 μC cm^–2^ and coercive fields (*E*_c_) of 1.67 and 2.00 kV cm^–1^ were recorded for **1** and **2**, respectively (Figure 3a,c). The origin of polarization in **1** and **2** can be attributed to their stable charge-separated structures containing the phosphonium cations and the distorted tetrahedral anions as well as their cumulative nonclassical C–H···X interactions. Furthermore, the leakage current measurements on both of these samples exhibited peaks at the domain switching points, confirming the ferroelectric nature of their obtained *P–E* loops. The slightly higher current densities observed for both **1** and **2** can be attributed to relatively lower packing densities of their as-made compacted discs in comparison with traditional sintered ceramics. The fatigue measurements showed that the observed polarization values for both of these samples were largely retained for up to 10^6^ cycles (Figure 3b,d).

**Figure 4:**
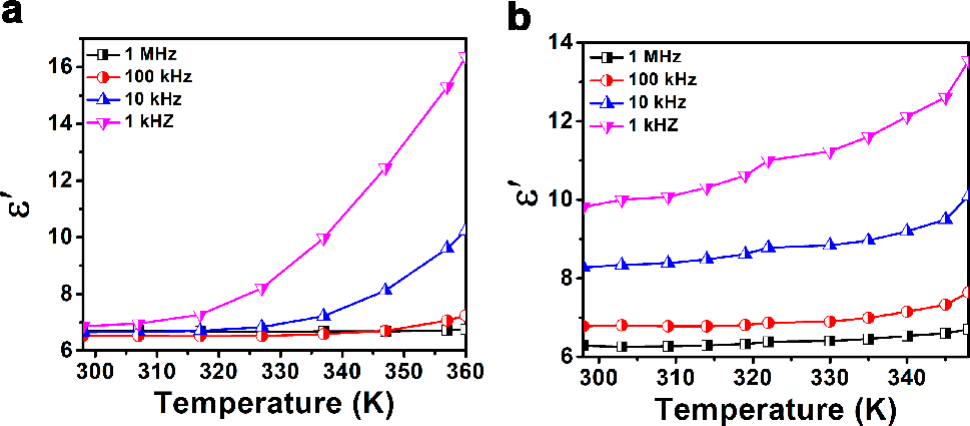
Temperature dependence of ε′ data for (a) **1** and (b) **2**.

The temperature- (*T*) and frequency (*F*)-dependent dielectric permittivity measurements were carried out for **1** and **2** on their powder pressed pellets. The real part of the dielectric permittivity (ε′) was initially found to increase linearly up to a certain temperature and was found to increase rapidly as the temperature approaches their corresponding melting points (Figure 4a,b). This is attributed to the presence of a large number of polarizable dipoles and mobile ions in those temperature regions. No Curie points (ferroelectric to paraelectric phase) were observed for both compounds during these temperature-dependent measurements. At 298 K and 1 MHz, the ε′ values were observed to be 6.71 and 6.20 for **1** and **2**, respectively. A small kink around 325 K in **2** could be attributed to the thermal motion of the dipoles during the volume expansion of the crystal. Also, the observed trends in tan δ versus *T* plots indicate a low dielectric loss (the dissipation of electrical energy in the form of heat) for both **1** and **2** (Figure S17a,b). Similar observations have been made in the ε′ versus *F* and tan δ versus *F* plots, as well, which indicates the involvement of all four polarization mechanisms in both **1** and **2** (Figures S18 and S19). The presence of electrical polarization in **1** and **2** is further supported by the ONIOM calculations, which gave a comparable dipole moment values for both these compounds (Table S4).

The direct piezoelectric response on the powder pressed pellets of **1** and **2** was measured with a piezometer system using “Berlincourt” method. The *d*_33_ values of 1.73 and 1.80 pC N^–1^ were recorded for **1** and **2**, respectively, at an operating frequency of 110 Hz and with an applied mechanical stress of 0.25 N.

### Fabrication and Characterizations of Phosphonium–TPU Composite Devices

**Figure 5:**
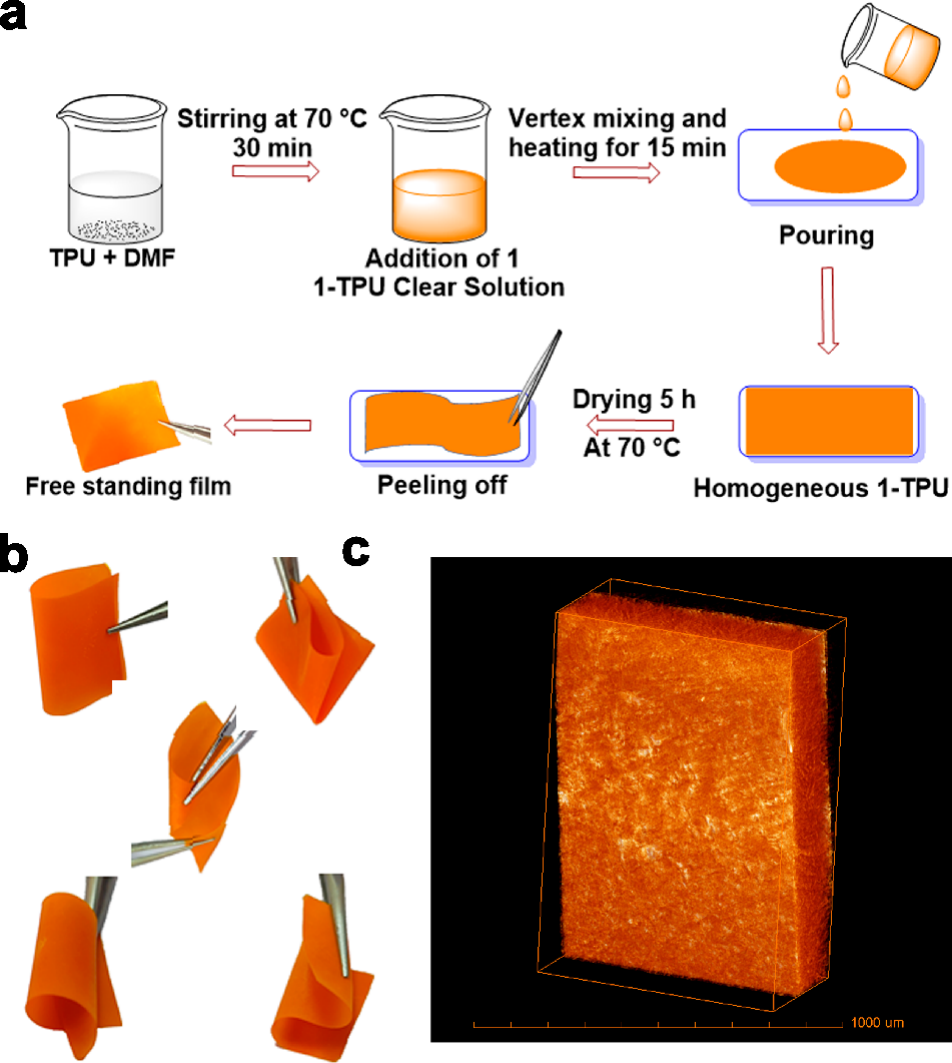
(a) Schematic diagram showing the preparation of **1**–TPU composite films. (b) Pictures of a representative **1**–TPU film showing its flexibility toward folding, bending, stretching, rolling, and two-fold bending operations (from top to bottom). (c) X-ray 3D microtomography images of 15 wt % **1**–TPU composite film (grid scale: 1000 μm).

Encouraged by the nanogenerator applications of earlier reported phosphonium salt–polymer composites, we examined these two halogenocuprate salts for mechanical energy harvesting applications. Unfortunately, polymer composites of **2** could not be prepared using TPU or any other polymer, presumably due to the reactivity of Cu–Br groups. Various (5, 10, 15, and 20) weight percent (wt %) composites of **1** with TPU have been prepared from a homogeneous solution of TPU and appropriate quantities of **1** in DMF as per the procedure outlined in Figure 5a. All polymer composite films of **1**–TPU were shown to exhibit excellent flexibility, as checked by application of various motions of folding, bending, stretching, rolling, and two-fold bending operations (Figure 5b).

**Figure 6:**
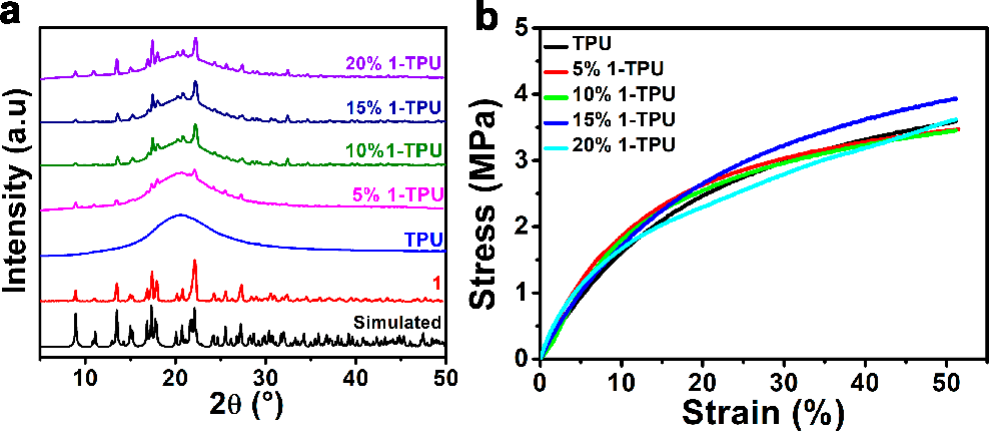
(a) Stacked PXRD profiles of **1**–TPU films and their comparison with the experimental and simulated diffraction patterns of **1**. (b) Stress–strain curves of TPU and **1**–TPU composite films.

The structural morphologies of the composite films were further visualized by the FE-SEM technique, which shows the uniform distribution of the ferroelectric particles in the composite films for up to 15 wt % of **1** (Figure S20a–d). However, agglomeration of the particles was evident for the composite with 20 wt % of **1**. Furthermore, X-ray 3D microtomography images were recorded for the optimal 15 wt % composite, which confirms the crystalline nature of the embedded particles in the polymer matrix (Figure 5c and Figure S21). The phase purity of **1** in the polymer–composite films was confirmed from the PXRD analysis. From the PXRD profiles, it is apparent that the crystalline behavior of the composite films improves as the wt % of **1** is increased from 5 to 20% (Figure 6a). The presence of randomly oriented crystallites in the TPU matrix is further evident from the presence of characteristic *hkl* peaks in the **1–**TPU composite films, which are present in the bulk powder sample of **1** (Figure S22).

Furthermore, the stress–strain relationships for all **1**–TPU composite devices were tested using a universal testing machine. These measurements, performed as a function of strain, show that all of the composite films are stretchable up to a 50% strain. The stress values, a measure of load bearing ability, at 50% strain were measured to be 3.58, 3.46, 3.44, 3.92, and 3.60 MPa for the neat TPU and 5, 10, 15, and 20 wt % **1**–TPU, devices, respectively (Figure 6b).^57^ A higher stress value observed for the 15 wt % **1**–TPU over its 20 wt % film could possibly indicate the agglomeration of the particles in the latter composite. The fact that the stress values for the composites at 50% strain were uncompromised relative to neat TPU suggests better structural and interfacial interaction between the polymer and the ferroelectric particles.

### Mechanical Energy Harvesting Outputs of the Composite Devices of **1**

**Figure 7:**
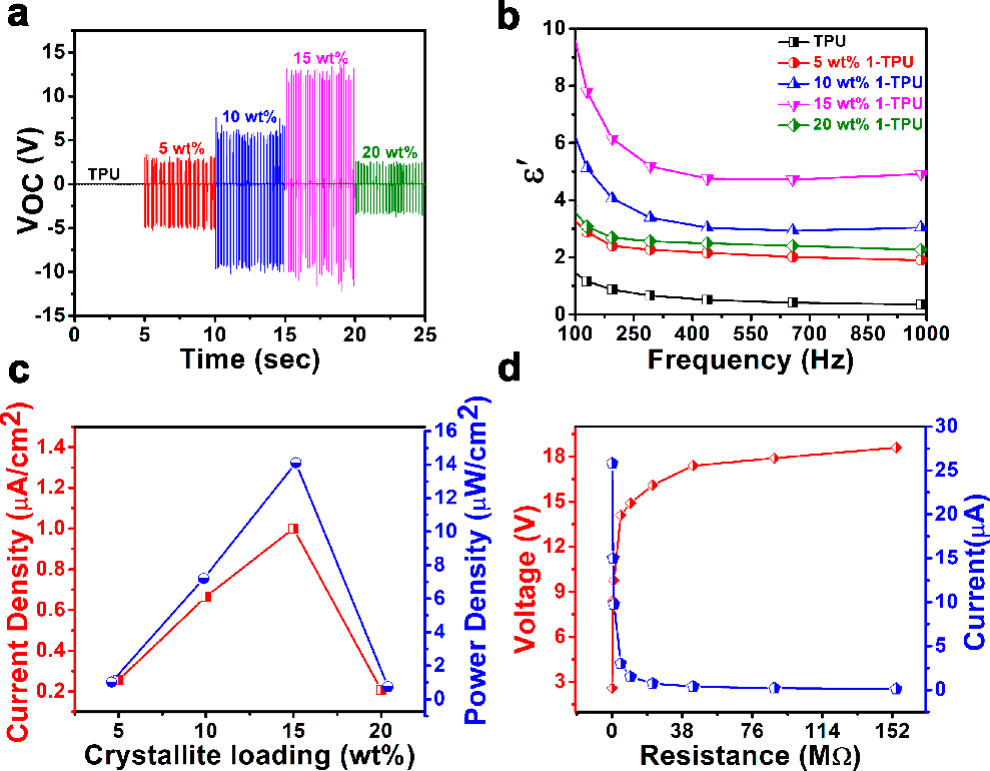
(a) Open-circuit output voltages of **1**–TPU composite devices. The shifted time axis provided here is a guide for the eye. (b) Frequency dependence of ε′ data for neat TPU and all **1**–TPU (5, 10, 15, and 20 wt %) composite films. (c) Current density and power density values of the **1–**TPU composite devices. (d) Comparative output voltage and calculated current data for the 15 wt % **1**–TPU composite device under various load resistances.

To perform the nanogenerator measurements, adhesive copper tapes along with contact leads (Cu wires) were placed on either side of the composite films. These electrode-deposited films were encapsulated in PDMS to protect them from the surface static charges, which are generated due to the application of external stress during the measurement. The piezoelectric characteristics of all of the devices were tested using a custom-built impact force setup coupled with an oscilloscope. All measurements involving **1**–TPU composite devices were performed in their nonpoled state with an optimized external load of 14.15 N and a frequency of 10 Hz. For details pertaining to the optimization, please see Figures S23 and S24. The peak-to-peak output voltages (*V*_PP_) were 7, 16, 25, and 5.4 V for 5, 10, 15, and 20 wt % **1**–TPU composite devices, respectively (Figure 7a). The short-circuit peak-to-peak currents (*I*_PP_) were measured to be 0.92, 2.4, 3.6, and 0.75 μA, respectively, for the 5, 10, 15, and 20 wt % **1**–TPU devices at an external resistance of 4.7 MΩ (Figure S26). By contrast, a device made up of neat TPU, under identical conditions, yielded an output voltage (*V*_PP_) of only 0.2 V (Figure 7a). This observation confirms that the piezoelectric response in the composite devices originates from the contribution of ferroelectric crystallites of **1** embedded in the films.

It is evident that the *V*_PP_ and *I*_PP_ profiles show an increasing trend with the rise in the percentage loading up to 15 wt % and decrease significantly for the 20 wt % **1**–TPU device. The reduction in the output performance of the 20 wt % devices is attributed to the agglomeration of the particle, which results in the randomization of the dipoles responsible for the piezoresponse of the materials.^11^ To verify this, the dielectric constant values of all of the composite films were measured at different frequencies (Figures 7b and S29a,b). The ε′ versus *F* profile showed an increase in ε′ value with the increase in loading of **1** onto the polymer matrix up to 15 wt %, and on further increasing the load to 20 wt %, a decrease in the ε′ values were observed. This indicates that the agglomeration of the crystallites results in the reduction of the dipole moments responsible for the polarization. A similar trend was also observed for the calculated current density and power density values, as well. A maximum current density value of 1.0 μA cm^–2^ and power density value of 14.1 μW cm^–2^ were observed for the optimal 15 wt % **1**–TPU composite device (Figure 7c).

To check the practical utility of the composite devices for energy harvesting applications, all **1**–TPU devices were tested under different external load resistances.^58^ The voltage outputs collected for each of the devices in the range of resistances from 0.1 to 157 MΩ showed a clear increase in the voltage outputs until a threshold resistance of 157 MΩ, beyond which it saturates (Figures 7d and S30a). For resistances below 0.1 MΩ, the voltage signals were not detected, which can be attributed to the high internal resistance of the composite devices (∼22 MΩ).^59^ To the best of our knowledge, the observed output performance of the 15 wt % **1**–TPU device is better than several of the earlier reported polymer composites of all-organic, and organic–inorganic hybrid perovskite materials (Table S5).^7,9,29,39,40,46,48,60−63^

Furthermore, the durability of 15 wt % **1**–TPU composite device was checked after a period of 3 months. The measurement showed no voltage drop even after 3 months, and the mechanical durability of this device was found to be intact (Figure S31). The fatigue measurements on the 15 wt % **1**–TPU composite device showed significant durability and retention of output voltage even after 10000 cycles with continuous application of 14.15 N external impact (Figure S32).

**Figure 8:**
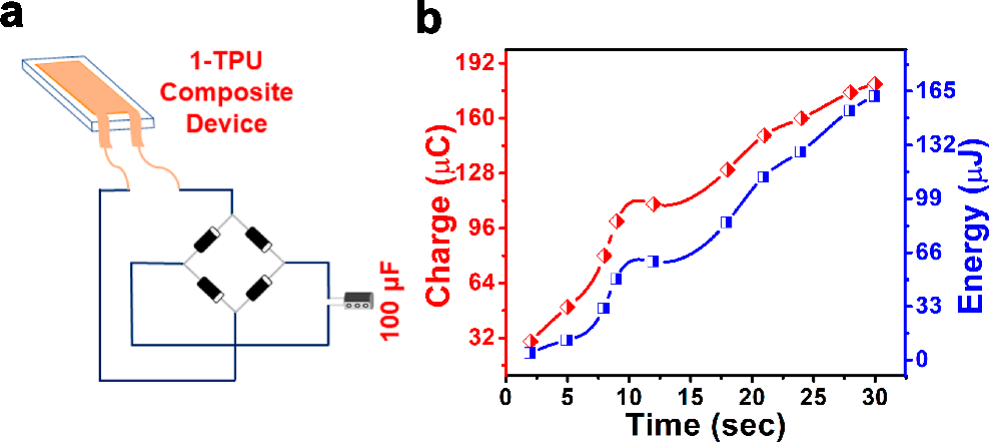
(a) Circuit diagram of the full-wave four diode bridge rectifier circuit. (b) Energy (*E*) and charge (*Q*) stored in the 100 μF capacitor. Lines are drawn to guide the eyes.

Driven by the excellent nanogenerator output performance, the 15 wt % **1**–TPU composite was subjected to the capacitor charging experiments by converting the AC output signals to the DC voltages using a full-wave four diode bridge rectifier circuit (Figure 8a). Using a 100 μF capacitor, the maximum stored voltage was found to be 1.8 V, which was attained within 30 s of applying the external force of 14.15 N (Figure S33). The disparity observed between the voltage stored in the capacitor and obtained *V*_PP_ can be attributed to the loss of voltage in the rectification process or the leakage of energy stored in the capacitor. The maximum stored energy (*E*) and measured charge (*Q*) in the capacitor are found to be 162 μJ and 180 μC, respectively (Figure 8b).

## Conclusion

To summarize, two discrete hybrid halogenocuprates **1** and **2** were synthesized and examined for ferroelectric and piezoelectric properties. The *P*–*E* hysteresis loop measurements on **1** and **2** gave a *P*_r_ value of 17.16 and 26.02 μC cm^–2^, respectively, which are higher than some of the reported organic–inorganic hybrid ferroelectric materials. The absence of any anomalous peaks in the temperature-dependent dielectric measurements confirmed the absence of any ferroelectric to paraelectric phase transitions. Polymer composites of various wt % of contributions of **1** were prepared with TPU, and their utility was evaluated for nanogenerator applications. These measurements gave a maximum output voltage of 25 V and power density of 14.1 μW cm^–2^ for the optimal 15 wt % **1**–TPU device. The output voltages generated from this device were further shown to rapidly charge a capacitor within 30 s. The durability of this device was found to be excellent even after a resting period of 3 months. These findings pave the way for the development of less toxic and less expensive ferroelectric nanogenerators based on 3d-metal ions for use in future wearable electronics.

## Supporting Information Available

Additional figures pertaining to crystal structures, dielectric measurements, PXRD, TGA, SEM, X-ray 3D microtomography, and tables of bond lengths, bond angles, and piezoelectric device fabrication and analyses (PDF)
X-ray crystallographic data for **1** (CIF)
X-ray crystallographic data for **2** (CIF)

## Supplementary Material

mg1c00046_si_001.pdf

mg1c00046_si_002.cif

mg1c00046_si_003.cif
