# Supplementary material for: Ferroelectricity and Piezoelectric Energy Harvesting of Hybrid A2BX4-Type Halogenocuprates Stabilized by Phosphonium Cations
Source: ACS Mater Au. 2021 Nov 8;2(2):124–31. doi: 10.1021/acsmaterialsau.1c00046 (PMC9888644; doi:10.1021/acsmaterialsau.1c00046)
Supplement: Supplementary file 1 — mg1c00046_si_001.pdf [file mg1c00046_si_001.pdf]

Supporting information
Ferroelectricity and Piezoelectric Energy Harvesting of Hybrid A_2BX_4 type Halogenocuprates Stabilized by Phosphonium Cations

Supriya Sahoo,[†] Thangavel Vijayakanth,[†] Premkumar Kothavade,[§,‖] Prashant Dixit,[⊥] Jan K. Zaręba,[*,#] Kadhiravan Shanmuganathan,[*,§,‖] Ramamoorthy Boomishankar[*,†,‡]

[†]Department of Chemistry and [‡]Centre for Energy Science, Indian Institute of Science Education and Research (IISER), Pune, Dr. Homi Bhabha Road, Pune – 411008, India

[§]Polymer Science and Engineering Division, CSIR-National Chemical Laboratory, Dr. Homi Bhabha Road, Pune – 411008, India

[‖]Academy of Scientific and Innovative Research, Ghaziabad- 201002, India.

[⊥]PZT Centre, Armament Research and Development Establishment, Dr. Homi Bhabha Road, Pune – 411021, India

[#]Advanced Materials Engineering and Modelling Group, Wrocław University of Science and Technology, Wybrzeże Wyspiańskiego 27, 50-370 Wrocław, Poland

Table of contents

S.No.	Details	Page No.
1	Experimental Section	2
2	X-ray crystallographic information	3-5
3	Characterisations and Hirshfeld surface analysis data	6-9
4	SHG, Dielectric studies and dipole moment calculations	10-11
5	**1**-TPU composites and their Piezoelectric studies	11-18
7	References	19

Synthesis of 1

To a stirred ethanolic solution of [$MePPh_3$]Cl (1 g, 2.79 mmol), $CuCl_2$ (0.312 g, 1.39 mmol) in ethanol was added slowly.[1] The reaction mixture was further stirred for another 30 minutes at room temperature and was filtered through a thick pad of celite. The yellow-colored solution was kept for crystallisation at room temperature. Yellow crystals of **1** were obtained after 7 days. Yield: 88 %. Melting point: 440-446 K. ^{31}P NMR (162 MHz, CD_3OD): δ 21.57.

Synthesis of 2

To a stirred ethanolic solution of [$MePPh_3$]Br (0.7g, 2.230 mmol), $CuBr_2$ (0.15g, 1.115 mmol) in ethanol was added slowly.[2] The reaction mixture was further stirred for another 30 minutes at room temperature and was filtered through a thick pad of celite. The violet-colored solution was kept for crystallisation at room temperature. Violet crystals of **2** were obtained after 7 days. Yield: 91 % for **1** and **2,** respectively. Melting point: 403-410 K. ^{31}P NMR (162 MHz, CD_3OD) δ 21.57.

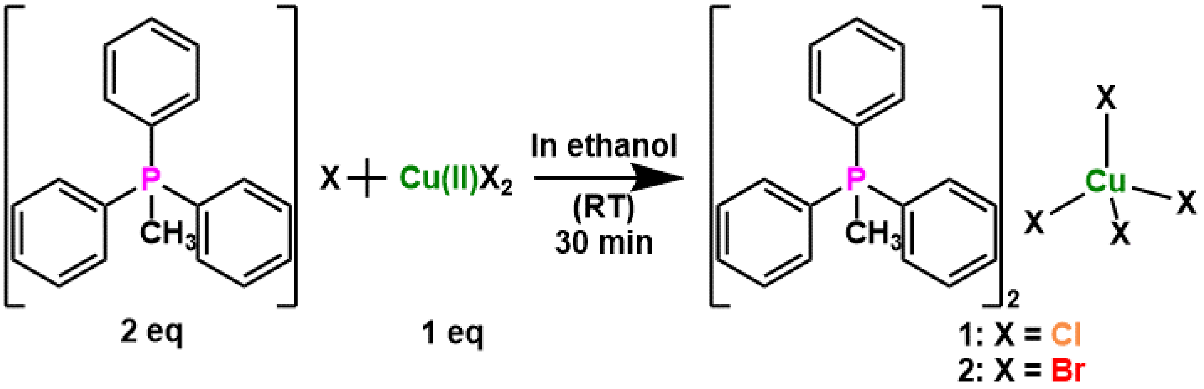

Scheme S1. Syntheses of **1** and **2**.

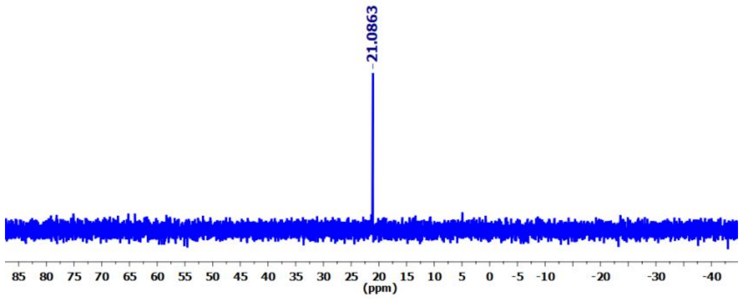

Figure S1. ^{31}P NMR of **1**.

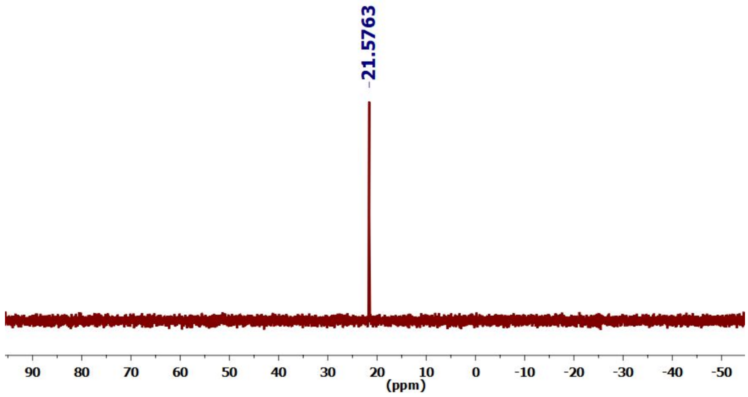

Figure S2. ^{31}P NMR of **2**.

Table S1. X-ray Crystallographic data for **1** and **2** at 100 K.

**Crystallographic details**	**1**	**2**
Chemical formula	$C_{38}H_{36}Cl_4CuP_2$	$C_{38}H_{36}Br_4CuP_2$
Formula weight (g/mol)	759.95	937.79
Temperature	100(2)K	100(2)K
Crystal system	Orthorhombic	Monoclinic
Space group	Fdd2	*Cc*
a (Å); α (°)	24.988(3); 90	11.9818(7); 90
b (Å); β (°)	32.738(4); 90	14.9270(8); 92.745(2)
c (Å); γ (°)	8.7611(9); 90	21.0066(11); 90
V ($Å^3$); Z	7167.1(13); 8	3752.8(4); 4
ρ (calc.) g cm^{-3}	1.409	1.660
μ(Mo K_α) mm^{-1}	1.024	4.952
$2\theta_{max}$ (°)	54.28	56.68
R(int)	0.0575	0.0369
Completeness to θ	100	98.8
Data / param.	3905/169	8715/348
GOF	1.046	1.096
R1 [F>4σ(F)]	0.0270	0.0215
wR2 (all data)	0.0667	0.0540
max. peak/hole (e.$Å^{-3}$)	0.363/-0.269	0.353/-0.506

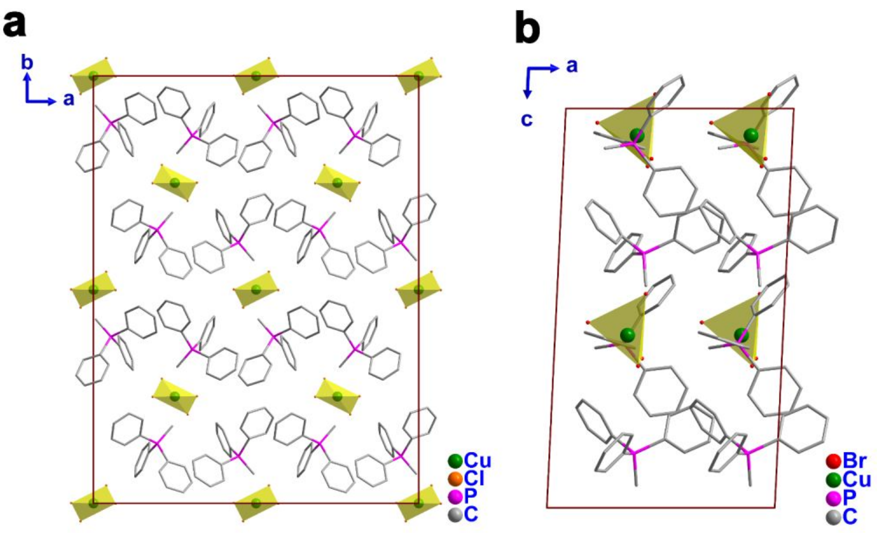

Figure S3. Packing diagrams of **1** (a) along c-axis and **2** (b) along b-axis at 100 K.

Table S2. Hydrogen bonding parameters for **1**.

D-H···A	d(H···A) Å	d(D-A) Å	<(DHA)	Symmetry transformations to generate equivalent atoms
C(34)-H(34)···Cl1	2.7808(23)Å	3.4492(72) Å	128.120(413)	x, y, z
C(36)-H(36)···Cl1	2.6608(25)Å	3.5395(71) Å	154.335(401)	1.25-x, -0.25+y, -0.25+z
C(1)-H(1B)···Cl1	2.7708(21)Å	3.6776(73) Å	153.674(403)	1.25-x, -0.25+y, -0.25+z
C(26)-H(26)···Cl2	2.6405(23)Å	3.4546(72) Å	144.016(406)	1-x, 1-y, z
C(1)-H(1A)···Cl2	2.6336(22)Å	3.5977(74) Å	167.343(465)	1-x, 1-y, z

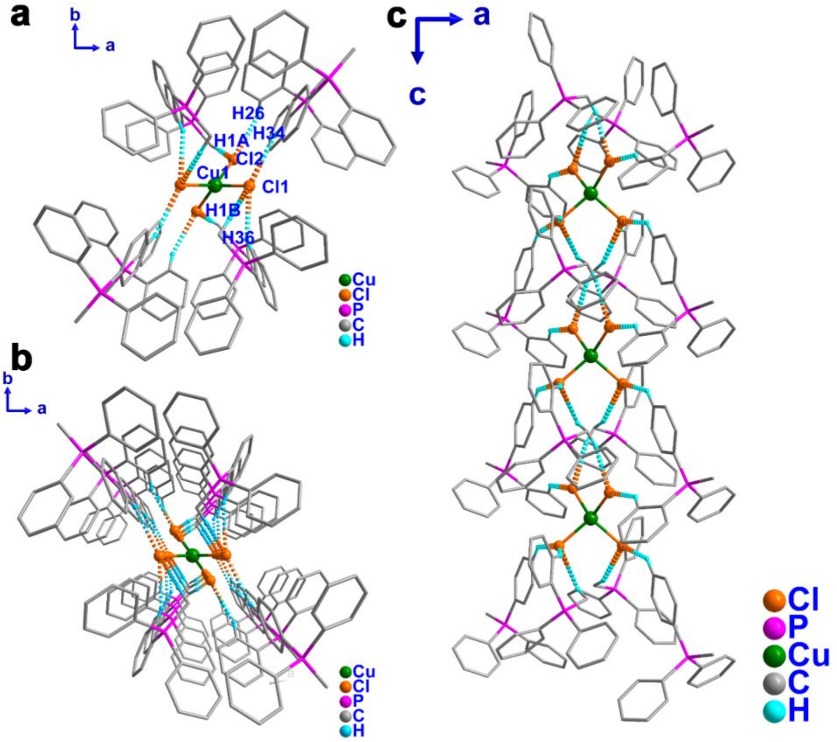

Figure S4. Non-classical C-H···Cl hydrogen bonding interactions in **1** at 100 K.

Table S3. Hydrogen bonding parameters for **2**.

D-H···A	d(H···A) Å	d(D-A) Å	<(DHA)	Symmetry transformations to generate equivalent atoms
C(46)-H(46)···Br4	2.8230(11)Å	3.5557(61) Å	1434.729(363)	x, y, z
C(22)-H(22)···Br1	2.7876(12)Å	3.7206(62) Å	166.771(370)	0.5+x, 0.5+y, z
C(2)-H(2C)···Br3	2.8683(11)Å	3.7483(70) Å	149.825(406)	0.5+x, 0.5+y, z
C(14)-H(14)···Br3	2.8124(10)Å	3.5403(82) Å	134.178(466)	x, 1-y, 0.5+z
C(25)-H(25)···Br3	2.8688(9) Å	3.5662(69) Å	131.109(412)	1+x, 1-y, 0.5+z

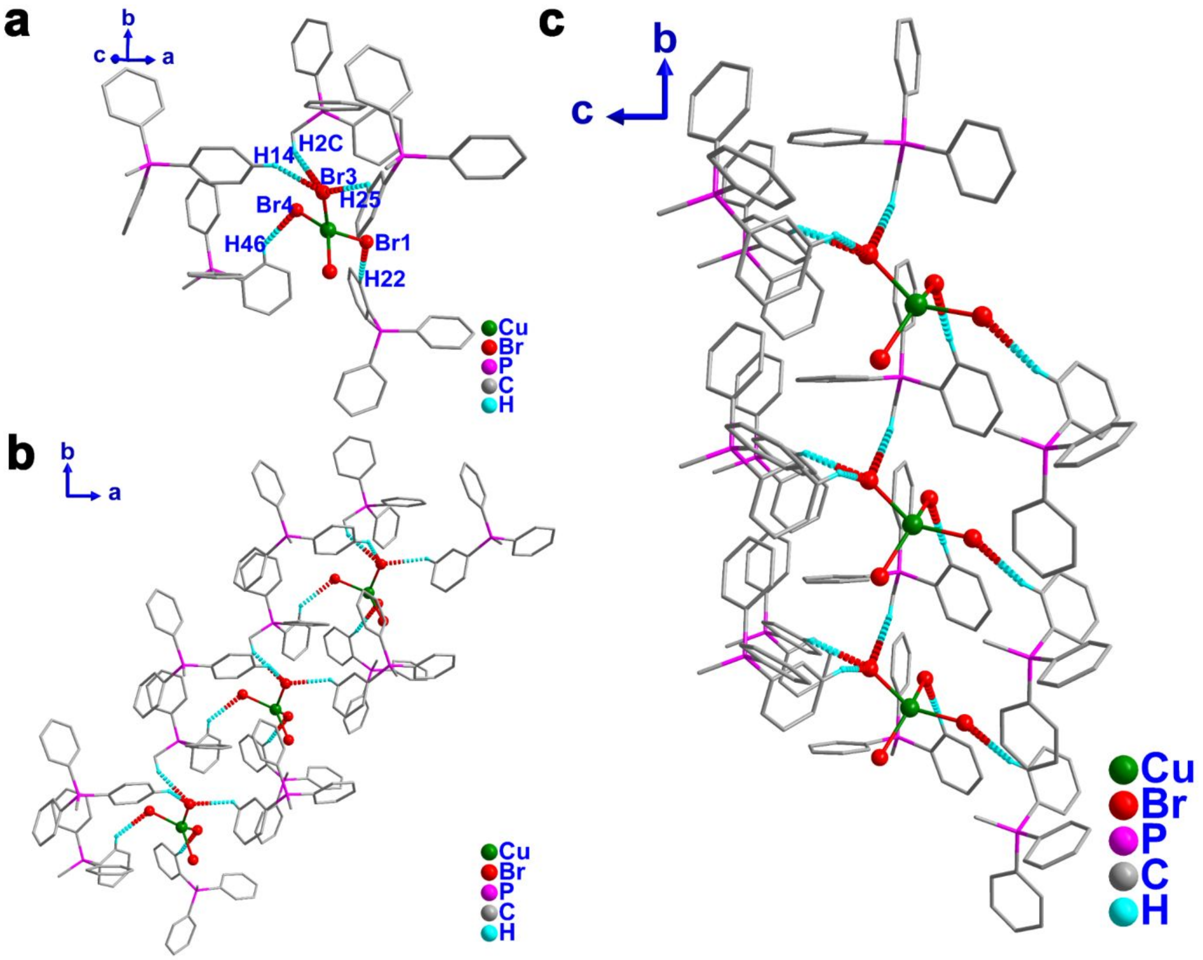

Figure S5. Non-classical C-H···Br hydrogen bonding interactions in **2** at100 K.

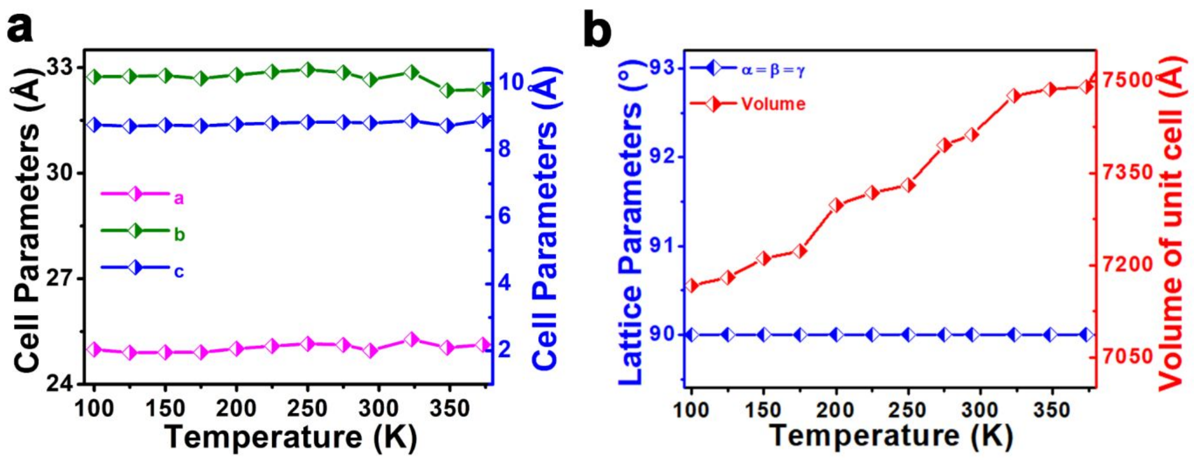

Figure S6. Variable temperature single crystal X-ray diffraction data of **1**.

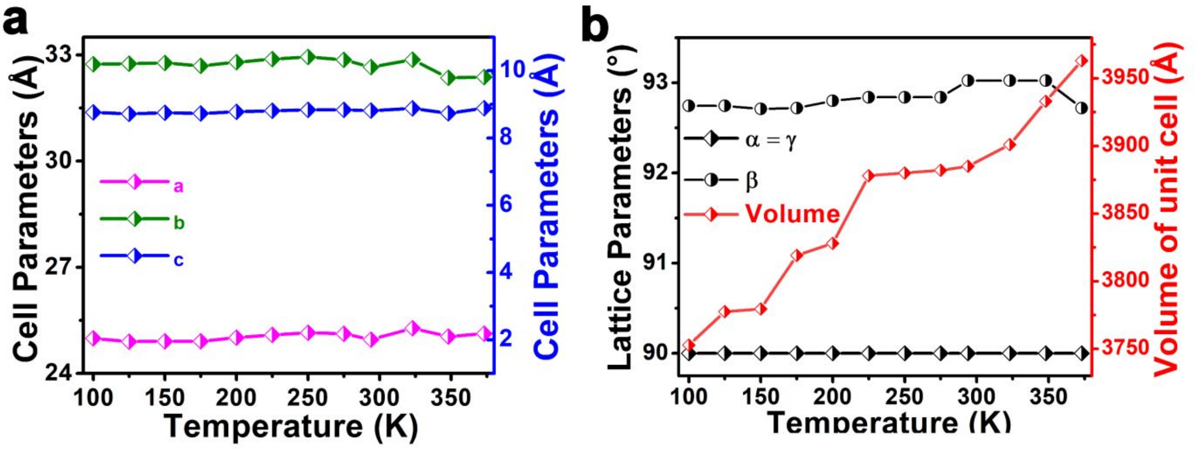

Figure S7. Variable temperature single crystal X-ray diffraction data of **2**.

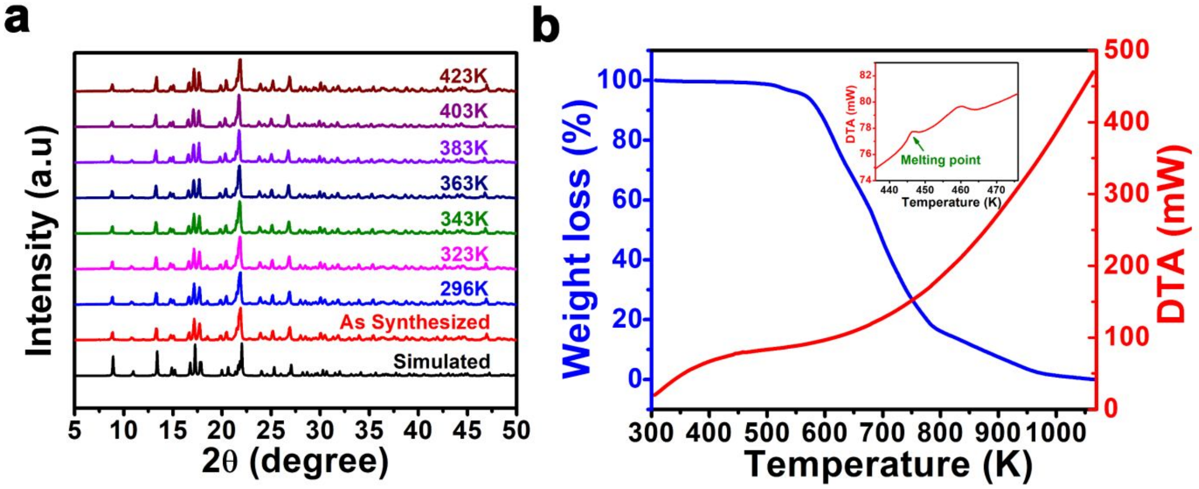

Figure S8. The (a) variable temperature powder X-ray diffraction and (b) thermogravimetric and differential thermal analysis profiles of **1**.

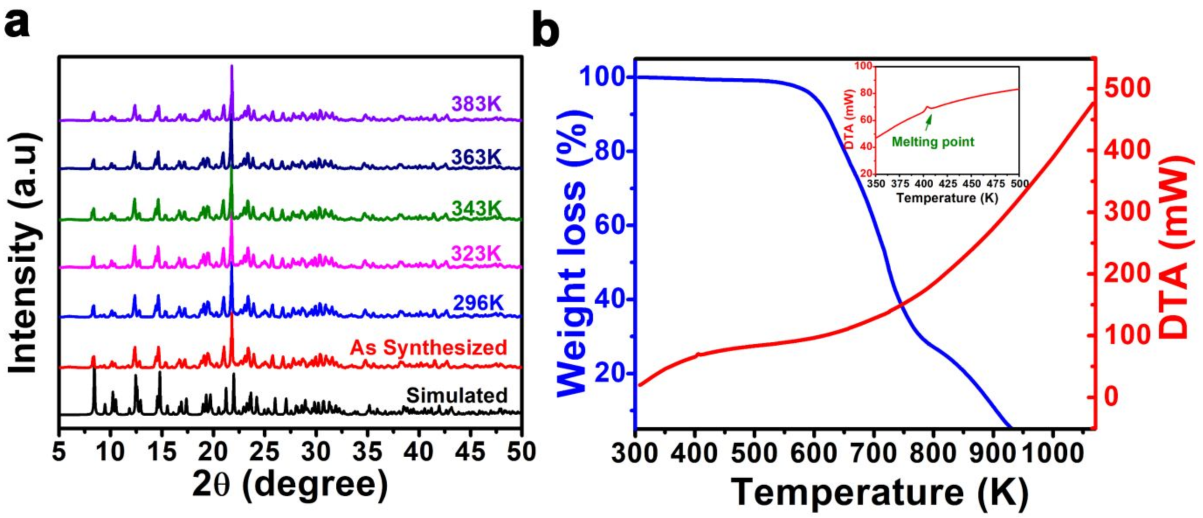

Figure S9. The (a) variable temperature powder X-ray diffraction and (b) thermogravimetric and differential thermal analysis profiles of **2**.

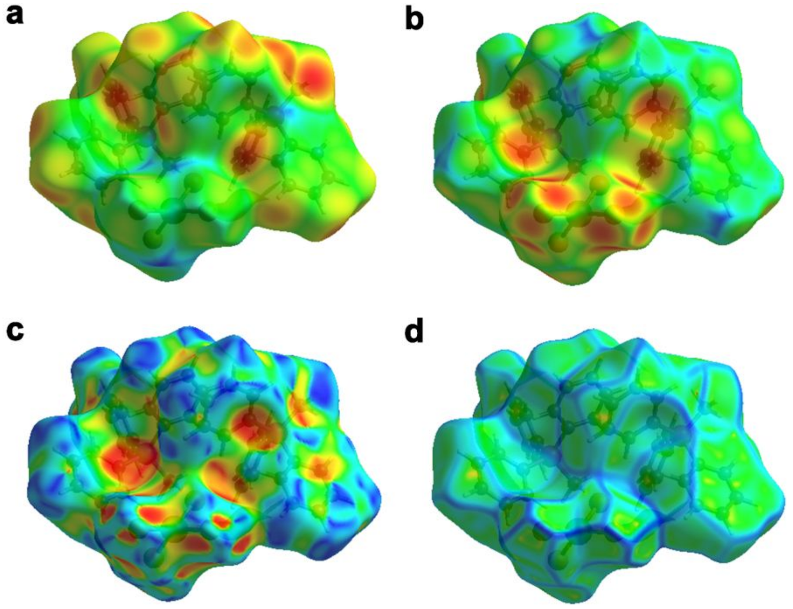

Figure S10. The 3D color mapping derived from the Hirshfeld surface analysis of **1** showing (a) d_i, (b) d_e, (c) shape index, (d) curvedness.

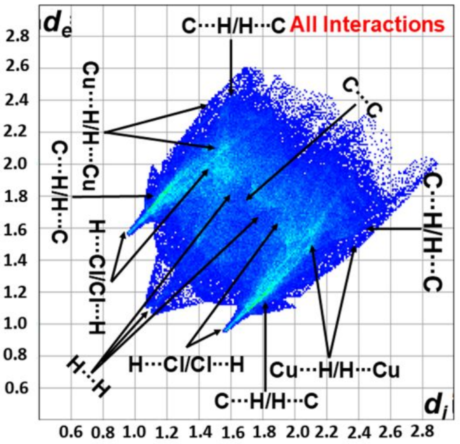

Figure S11. 2D fingerprint (d_e vs d_i) plot of **1** showing all the possible interactions in the molecule.

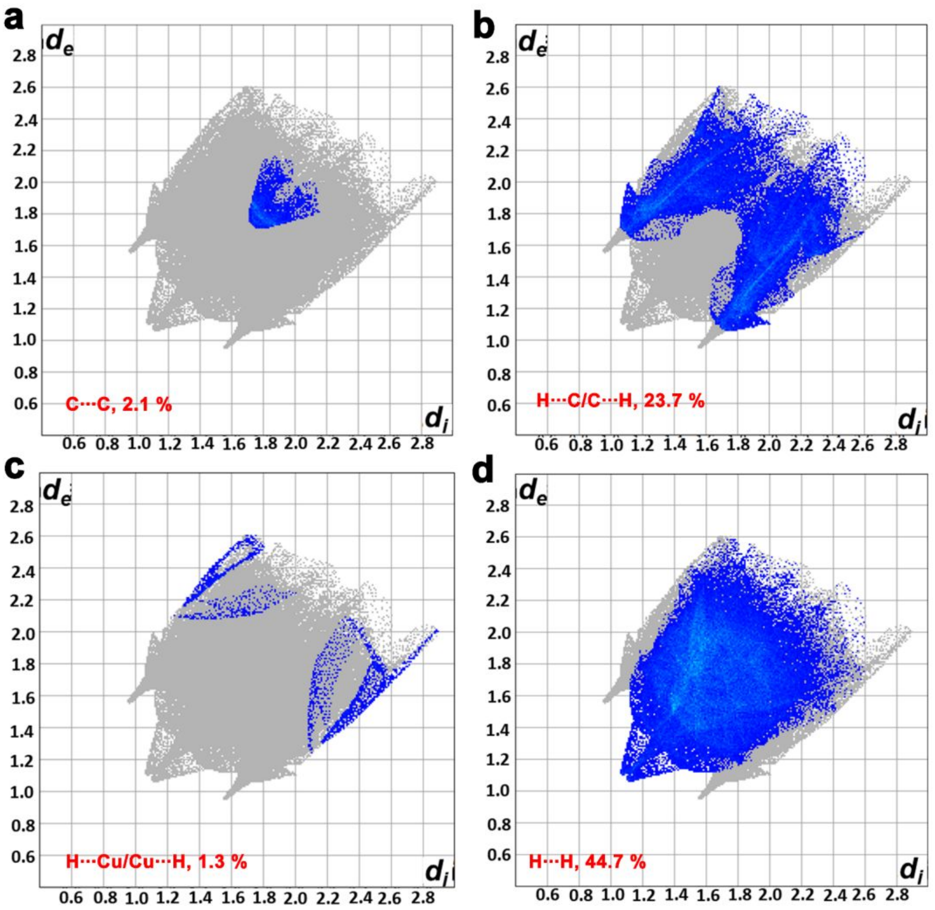

Figure S12. 2D fingerprint (d_e vs d_i) plot of **1** showing the percentages of (a) C···C, (b) C···H, (c) Cu···H, and (d) H···H interactions in the molecule.

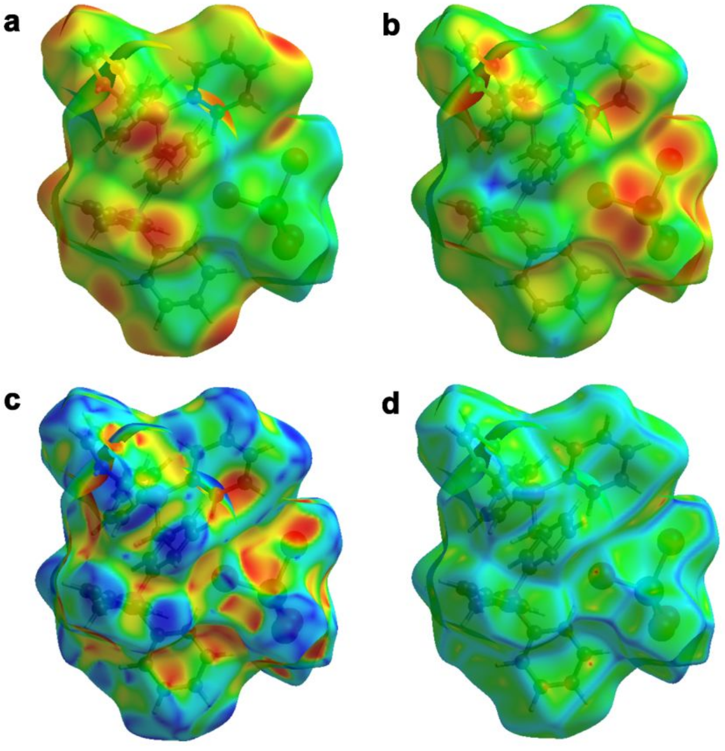

Figure S13. The 3D color mapping derived from the Hirshfeld surface analysis of **2** showing (a) d_i, (b) d_e, (c) shape index, (d) curvedness.

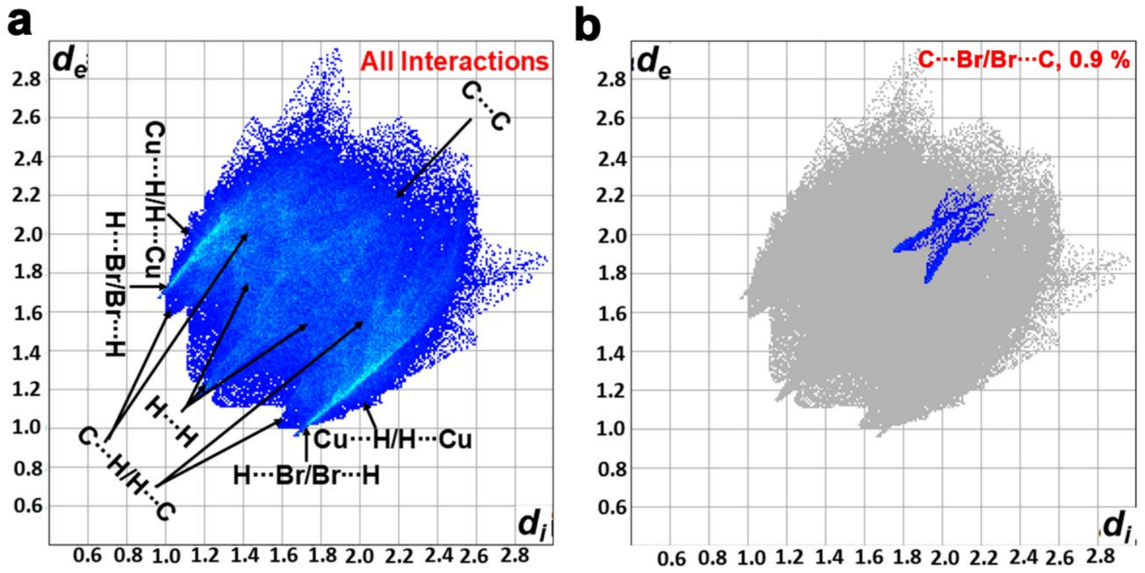

Figure S14. 2D fingerprint (d_e vs d_i) plot of **2** showing (a) all interactions in the molecule and (b) the percentage of C···Br interactions**.**

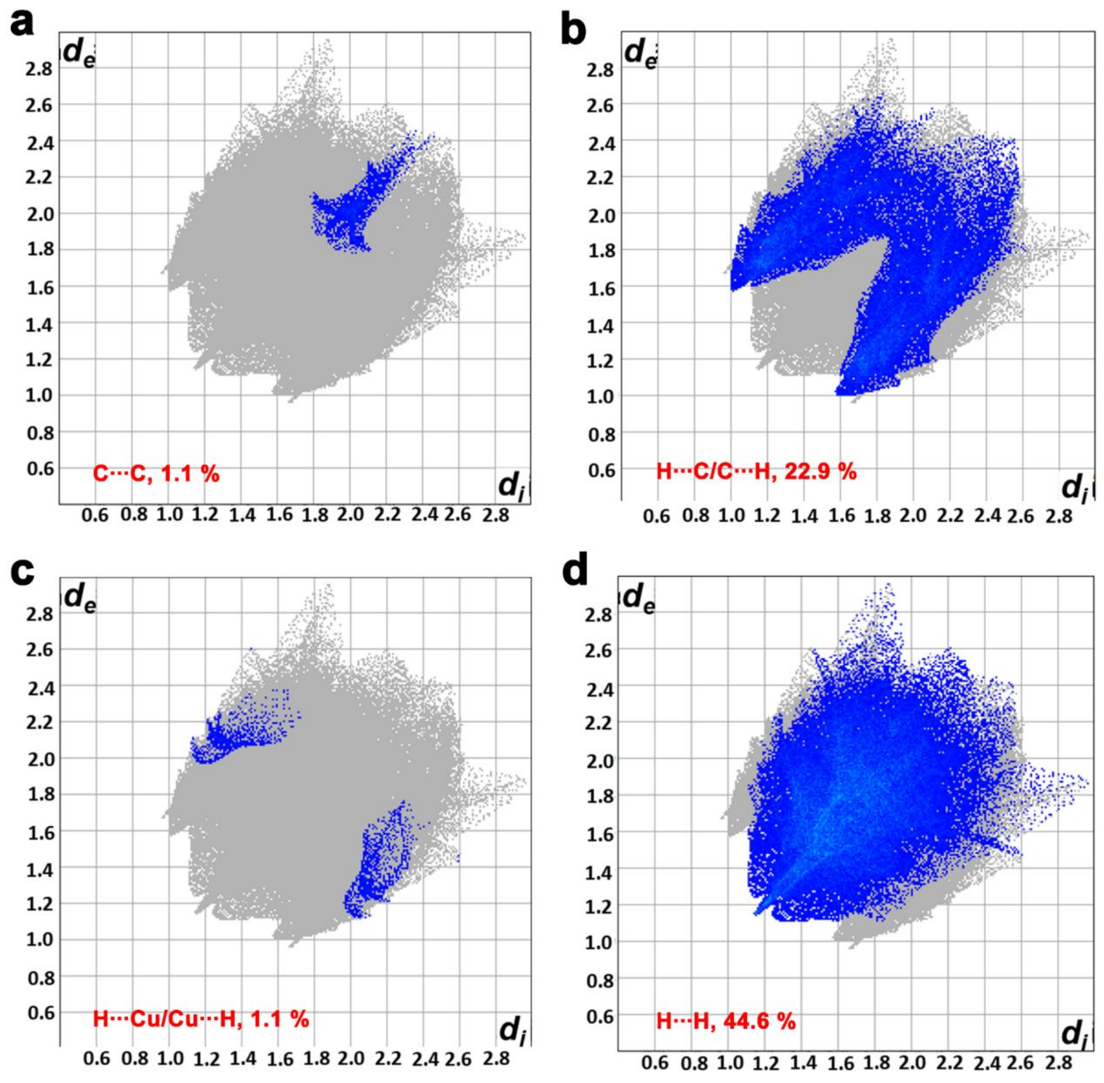

Figure S15. 2D fingerprint (d_e vs d_i) plot of **2** showing percentages of (a) C···C, (b) C···H, (c) Cu···H, and (d) H···H interactions in the molecule.

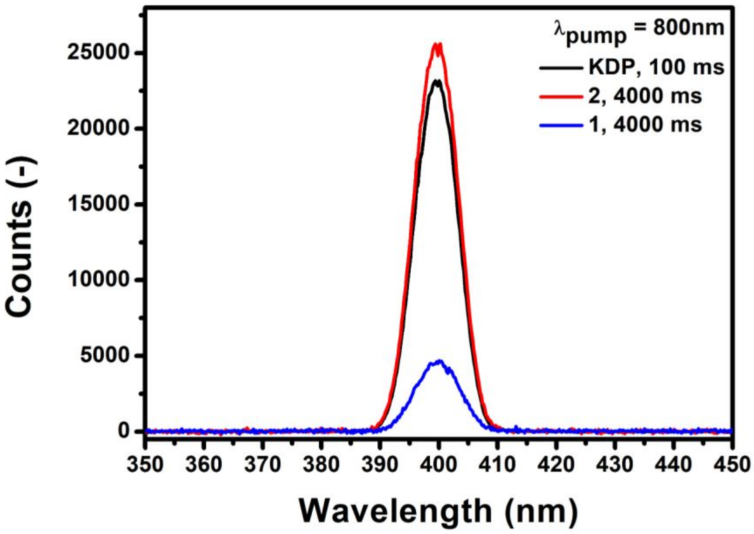

Figure S16. Spectra of second-harmonic generation (SHG) signals of **1** and **2** and their comparison with that of KDP**.** Note that signal intensities for **1** and **2** are not normalized to the same collection time of KDP for clarity of presentation.

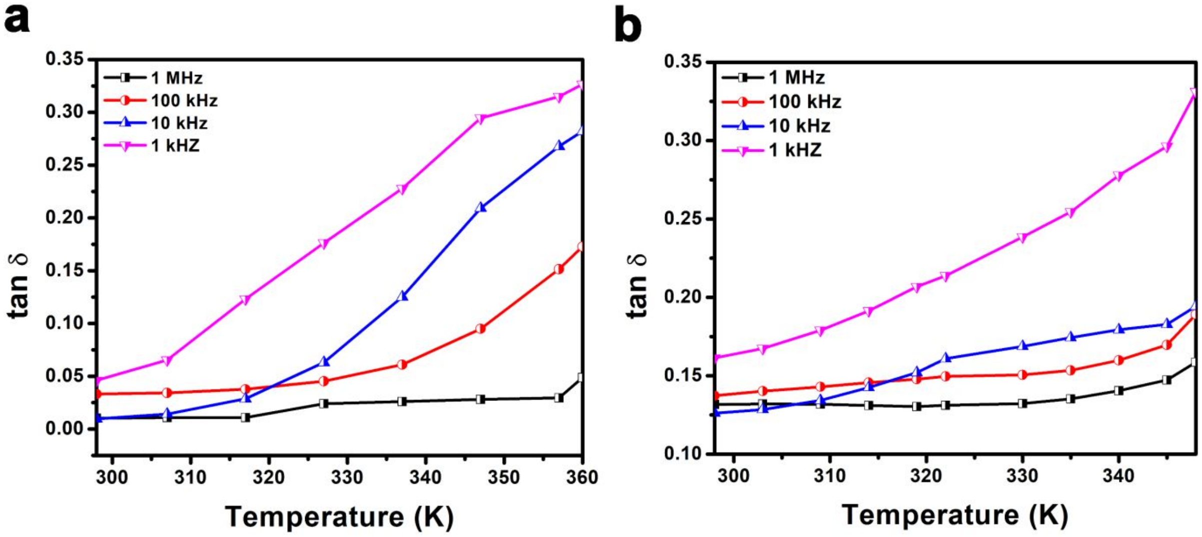

Figure S17. Temperature dependant dielectric loss plots of (a) **1** and (b) **2**.

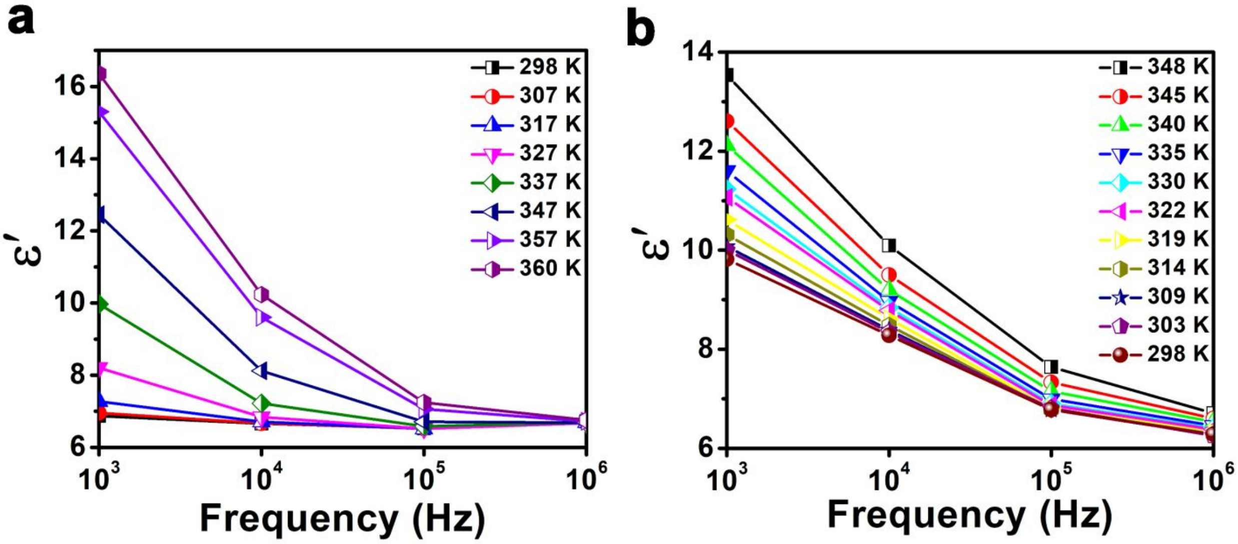

Figure S18. Frequency dependant dielectric permittivity plots of (a) **1** and (b) **2**.

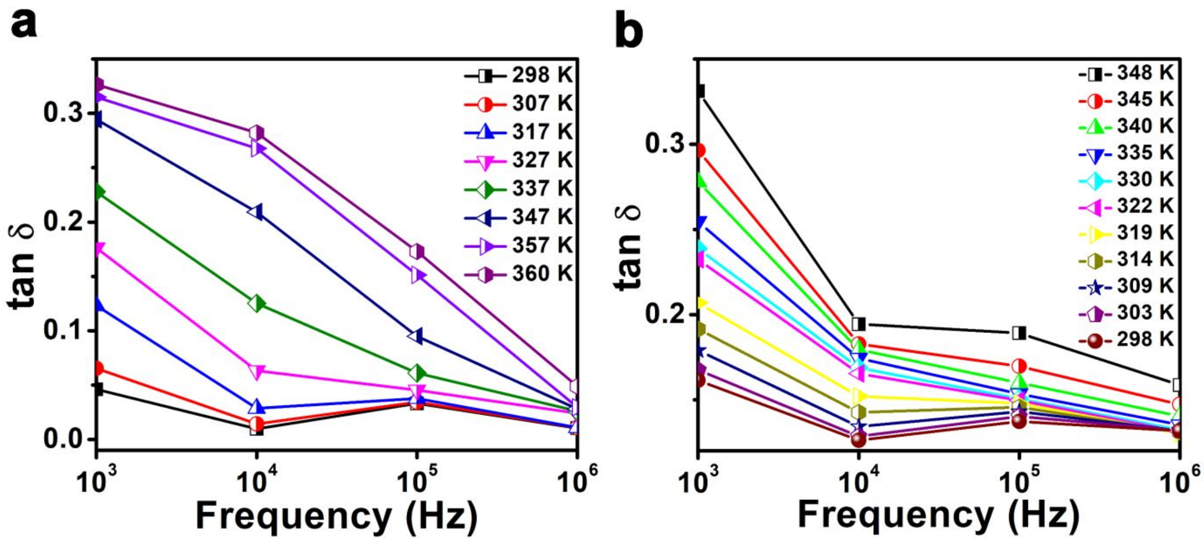

Figure S19. Frequency dependant dielectric loss plots of (a) **1** and (b) **2**.

Table S4. Dipole moment calculation of **1** and **2**.

Ferroelectric Material	Dipole moment in Debye unit (D)
**1**	39.8
**2**	31.3

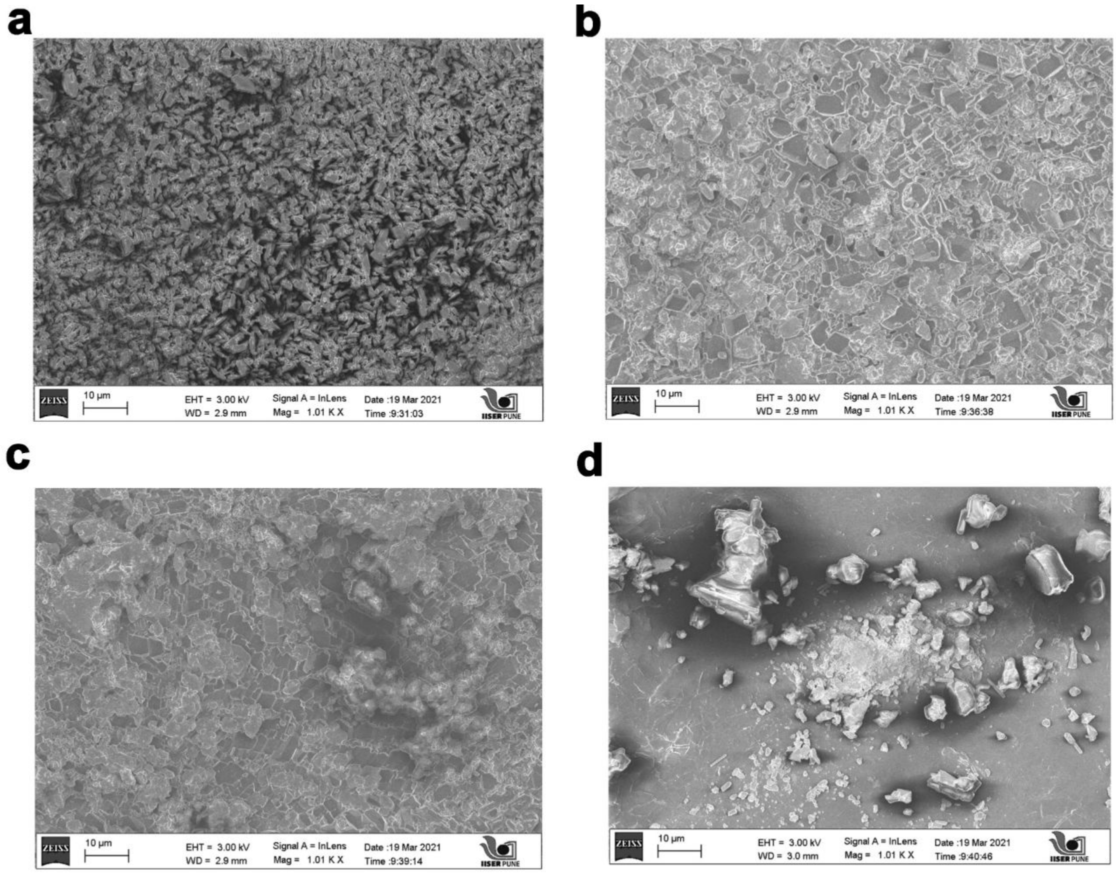

Figure S20. SEM images of **1**-TPU composites. The figures a, b, c, and d correspond to 5, 10, 15 and 20 wt % **1**-TPU composites, respectively.

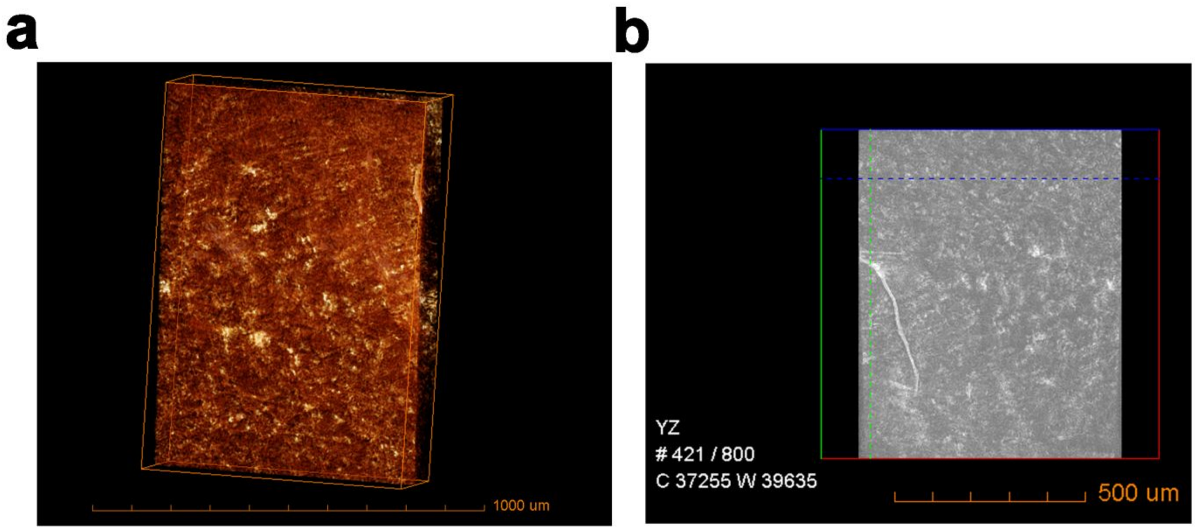

Figure S21. (a) X-ray 3D-microtomography (grid scale: 1000 μm, the polymer background was corrected to visualize the location of the crystallites of **1**) and (b) X-ray 2D-microtomography images of 15 wt % **1**-TPU composite film.

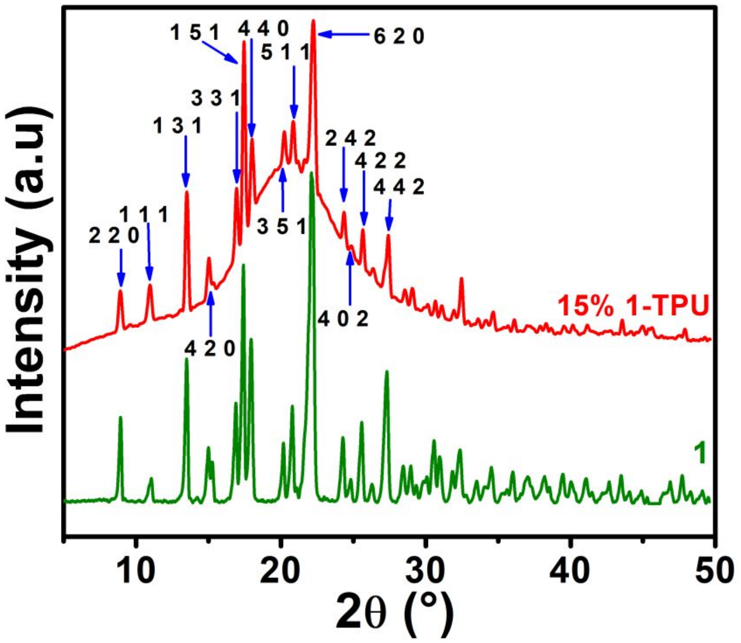

Figure S22. The powder X-ray diffraction pattern and the characteristic *hkl* peaks for compound **1** and 15 wt % **1**-TPU.

Impact force calculation

The impact force has been calculated using energy conservation law. The vertical rod has a potential energy, *PE* = *mgh* and kinetic energy, KE = 0 while it is at the top.

The kinetic energy just before impact is equal to its gravitational potential energy at the hight from which it was dropped.

PE = *KE*,

which means KE= *mgh* = 1/2mv^2 ……………………………………………(1)

So, the velocity just before the impact becomes,

v $= \sqrt{2gh}$……………………………………………………………………(2)

where, g = 9.81 m/s^2 is gravitational acceleration, m is the weight of falling vertical rod (130 g), and h is height (22 mm).

Substituting the values of m and h in the equation, we obtained the velocity, v = 0.66 m/s.

Substituting the values of velocity and the mass in the equation, the kinetic energy was calculated to be 0.02831 N.m.

For a straight-line impact collision, total work done is equal to average force of impact times the distance travelled (d) during the impact, which is ~2 mm in our case.

Average impact force = KE/d……………………………………………….(3)

Hence the, average impact force = 0.02831/0.002 =14.15 N.

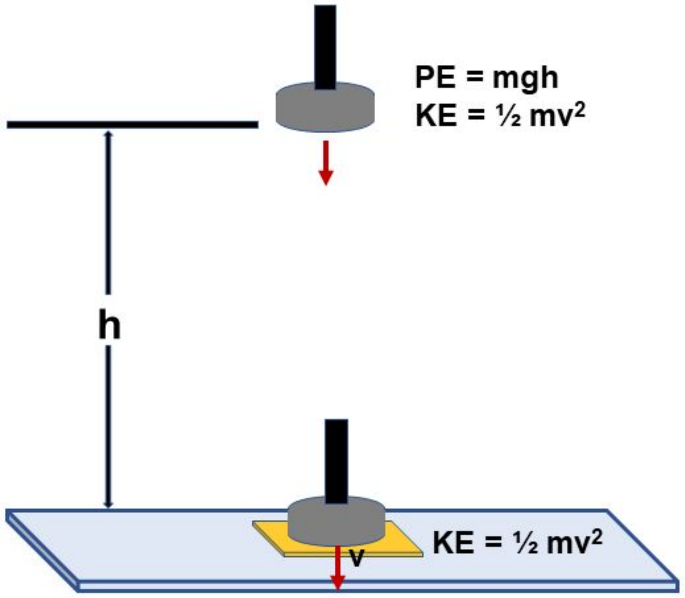

Figure S23. Schematic for calculation of impact force.

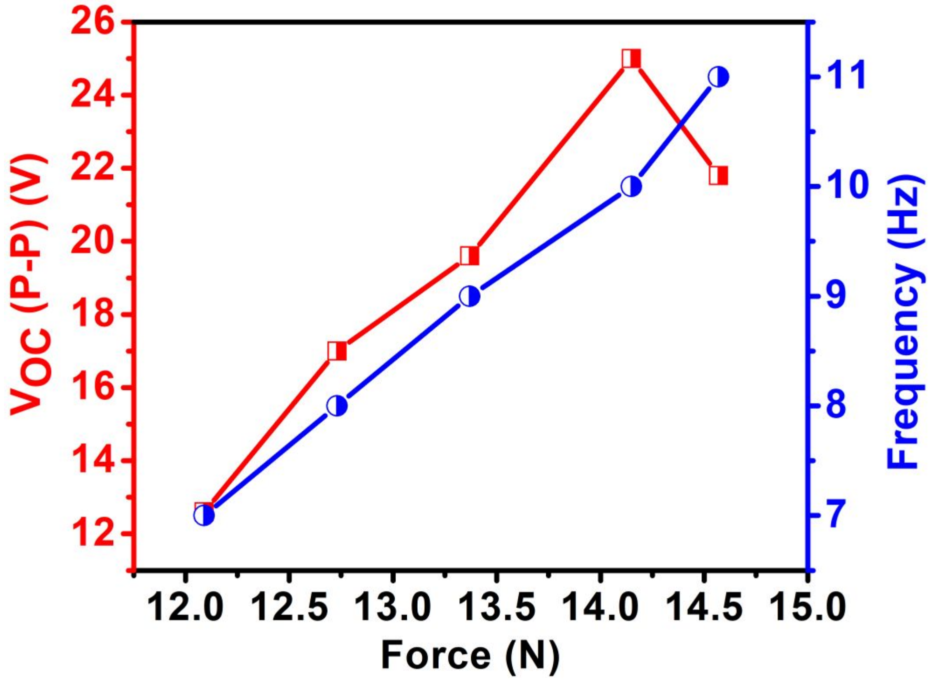

Figure S24. Observed trends in V_{PP} and frequencies of measurement which changes as a function of impact force for the 15 wt % **1**-TPU composite device.

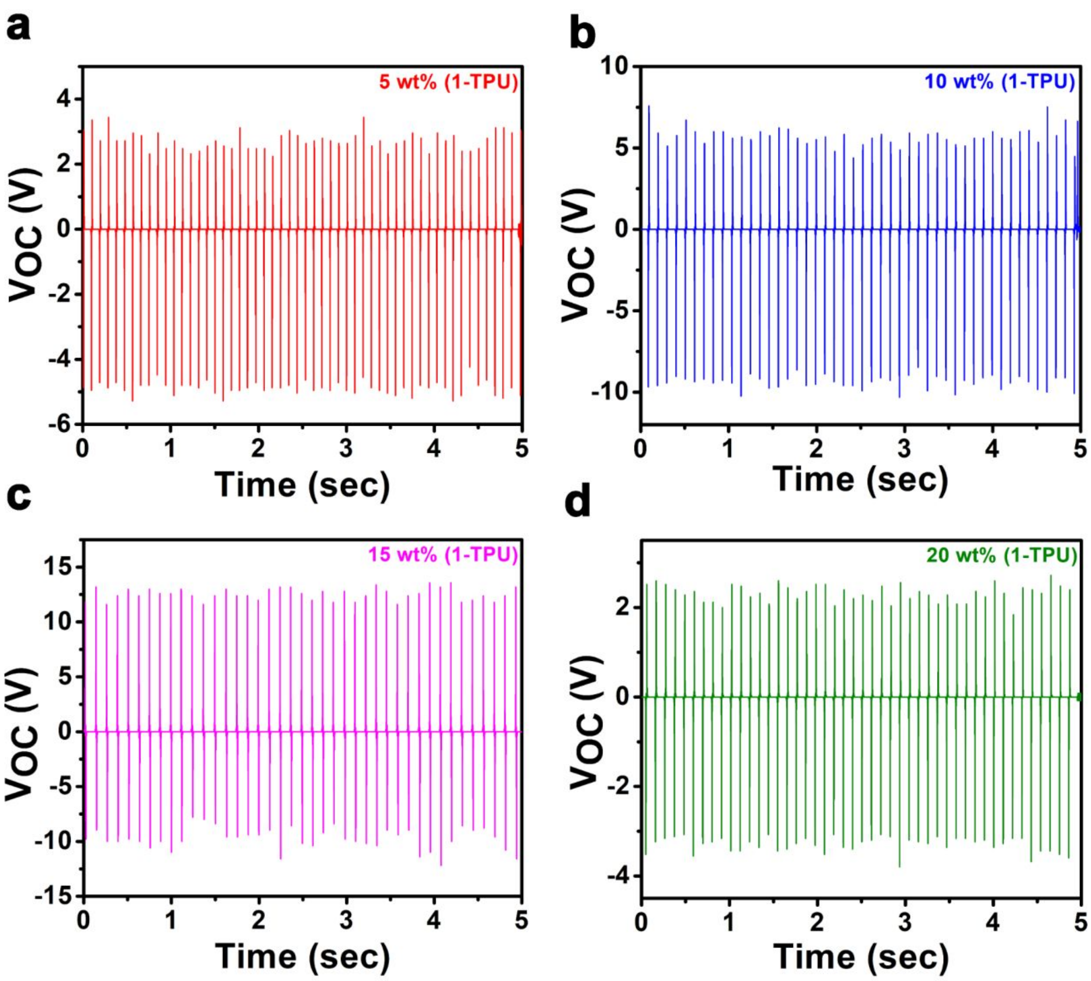

Figure S25. Output voltage profiles of **1**-TPU composite films.

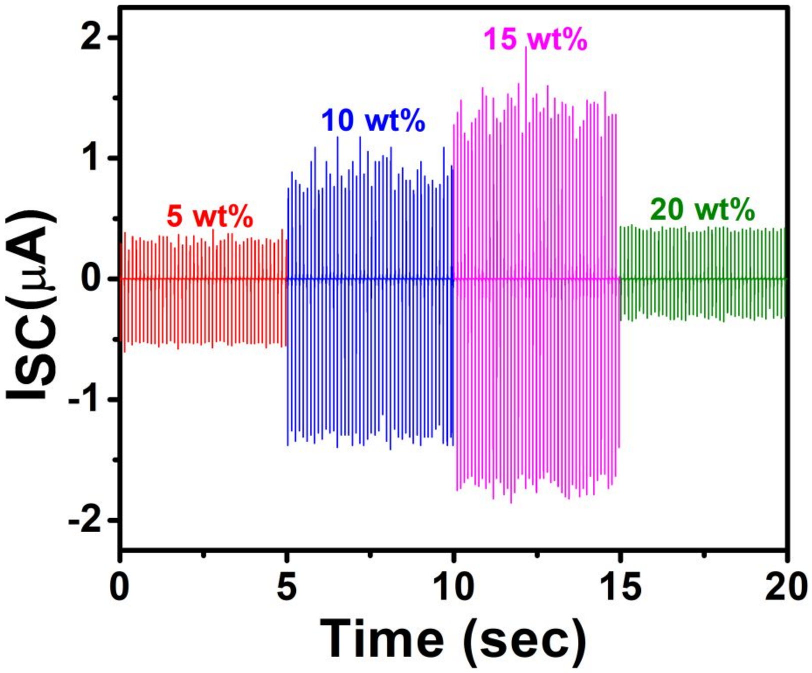

Figure S26. Calculated short-circuit currents of **1**-TPU composite devices. The shifted time-axis provided here is a guide to the eye.

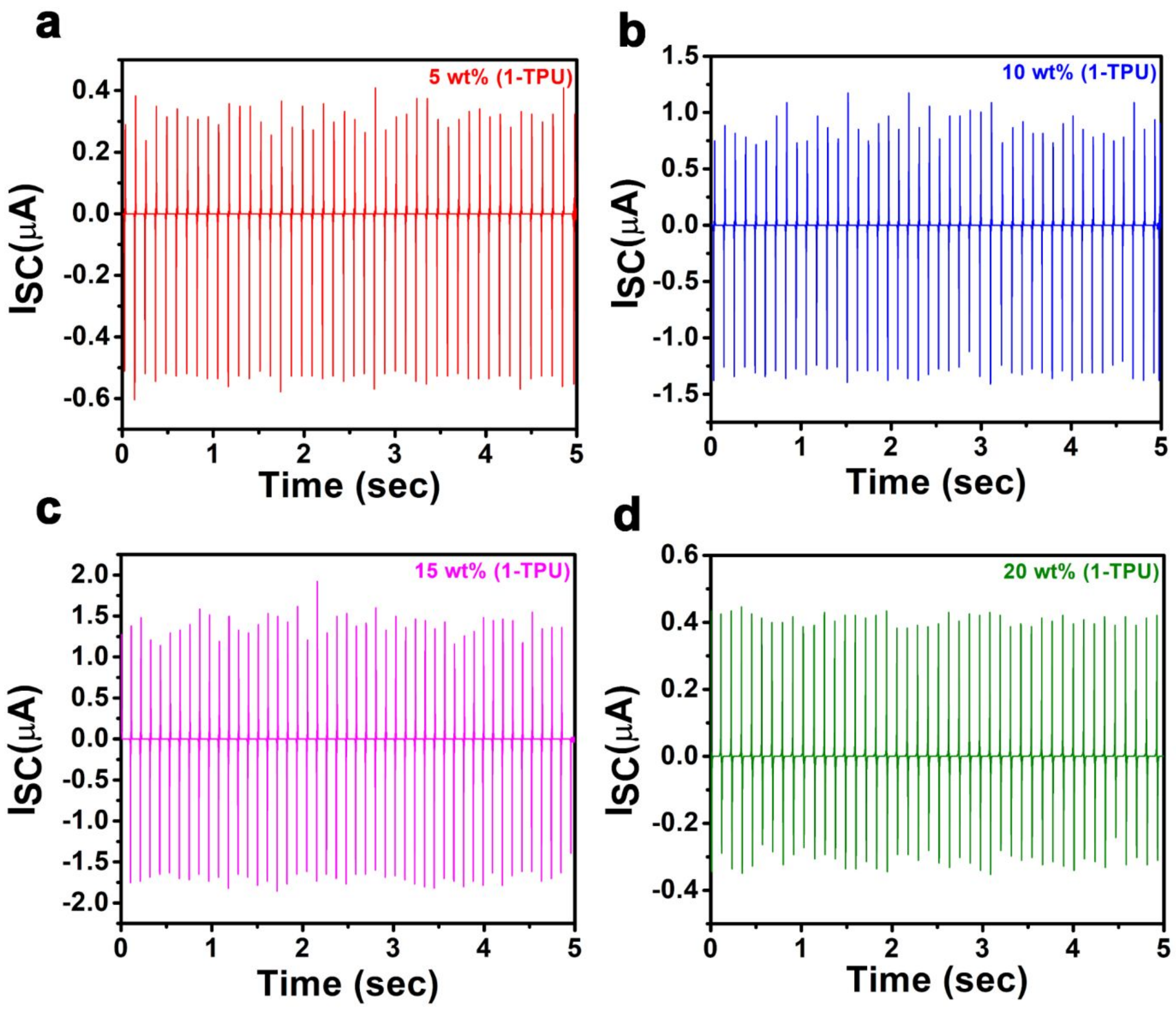

Figure S27. The calculated output currents of all **1**-TPU composite films.

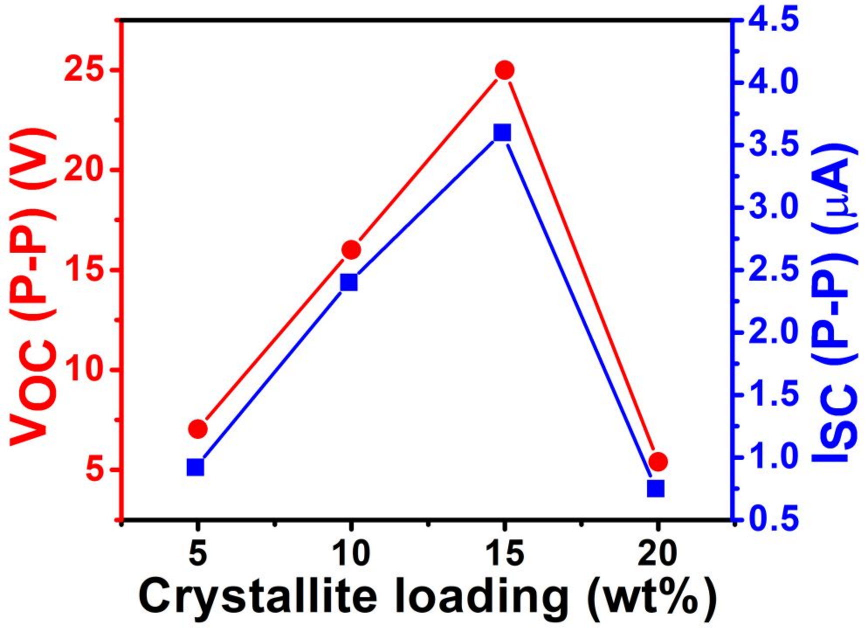

Figure S28. Comparative diagram showing the observed trends in V_{PP} and I_{PP} values of **1**-TPU composite devices.

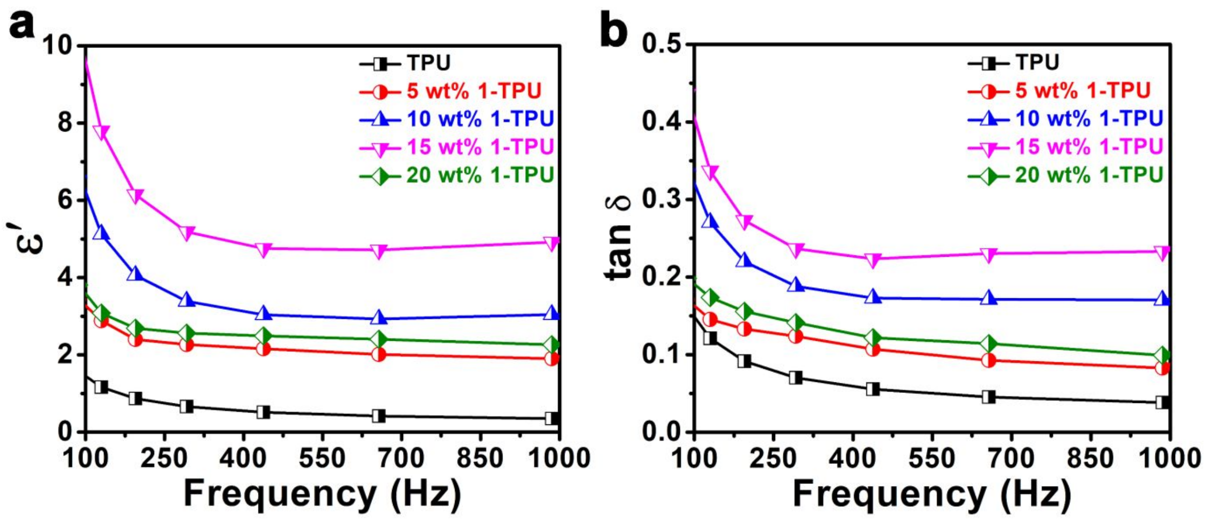

Figure S29. Frequency dependant (a) real-part of dielectric permittivity and (b) dielectric loss data for all **1**-TPU composite films.

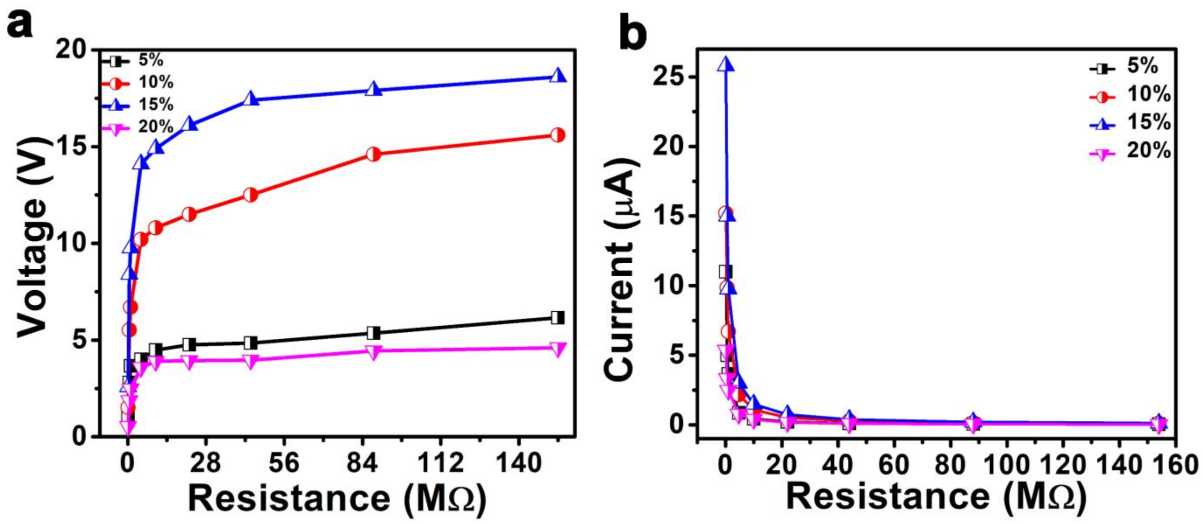

Figure S30. The comparative output (a) voltage and (b) current data for all the **1**-TPU composite devices under various load resistances.

Table S5. Comparison of output device performances of known composite energy harvesters.

Hybrid Composite Devices	Output Voltages	Current/Current density	Power/Power density	Active area	References
$MAPbI_3$-PVDF	9.43	0.76 μA cm^{-2}	-	1 x 1 cm^2	3
$MAPbBr_3$-PVDF	5	60 nA	0.28 μW cm^{-2}	2.4 x 1.5 cm^2	4
$MAPbI_3$-PDMS	1.0	50 nA cm^{-2}	-	1 x 1 cm^2	5
$FAPbBr_3$–PDMS	4	-	-	1 x 1 cm^2	6
$CsPbBr_3$/PVDF	10.3	1.29 μA cm^{-2}	3.31 μW	1 x 1 cm^2	7
PVDF–PLLA–SnO_2 NF–$MAPbI_3$	4.82	29.7 nA	-	0.25 x 0.25 cm^2	8
SnO_2 NF–$MAPbI_3$	1.02	10.32 nA	-	0.25 x 0.25 cm^2	8
$[BnNMe_3]_2CdBr_4$/PDMS	52.9	0.23 μA cm^{-2}	13.8 μW cm^{-2}	3 x 3 cm^2	9
$[BnNMe_2{}^nPr]_2CdBr_4$/PDMS	63.8	0.59 μA cm^{-2}	37.1 μW cm^{-2}	3 x 3 cm^2	9
(TMFM)$FeBr_4$	2.2	-	-	-	10
DPDP·PF_6/PDMS	8.5	0.28 μA cm-2	0.14 μW cm^{-2}	1.8 x 1 cm^2	11
DPDP·PF_6/TPU	6.73	0.12 μA cm^{-2}	0.06 μW cm^{-2}	1.3 x 3 cm^2	11
DPDP·BF_4/TPU	8.95	0.23 μA cm^{-2}	0.20 μW cm^{-2}	1.3 x 3 cm^2	11
TPAP·BF_4/TPU	7.37	0.16 μA cm^{-2}	0.09 μW cm^{-2}	1.3 x 3 cm^2	11
TIAP·BF_4/TPU	4.75	0.11 μA cm^{-2}	0.04 μW cm^{-2}	1.3 x 3 cm^2	11
TMAB	10	4.46 μA	-	-	12
$[Ph_3MeP]_4[Ni(NCS)_6]$/TPU	19.29	3.59 μA cm^{-2}	2.51 mW cm^{-3} (50.26 μW cm^{-2})	1.3 x 3 cm^2	13
**15 wt % 1-TPU**	**25**	**1.1 μA cm^{-2}**	**14.1 μW cm^{-2}**	**1.2 x 3 cm^2**	This work

Note: $MAPbI_3$ = methylammonium lead iodide; PVDF = polyvinylidene difluoride; PDMS = polydimethysiloxane; $FAPbBr_3$ = formamidinium lead bromide; PLLA = poly(L-lactic acid); SnO_2 = tin oxide; NF = nanofiber; $[BnNMe_3]_2CdBr_4$ = N,N,N-trimethyl-1-phenylmethanaminium cadmium(II) bromide; $[BnNMe_2{}^nPr]_2CdBr_4$ = N-benzyl-N,N-dimethylpropan-1-aminium cadmium(II) bromide; (TMFM)$FeBr_4$ = trimethylfluoromethylammonium iron(III)bromide; DPDP·PF_6 = diphenyl diisopropylamino phosphonium hexaflurophosphate; TPU = thermoplastic polyurethane; DPDP·BF_4 = diphenyl diisopropylaminophosphonium tetrafluoro borate; TPAP·BF_4 = triphenyl isopropylaminophosphonium tetrafluoro borate; TIAP·BF_4 = tetraisopropylaminophosphonium tetrafluoro borate; TMAB = trimethylamine borane.

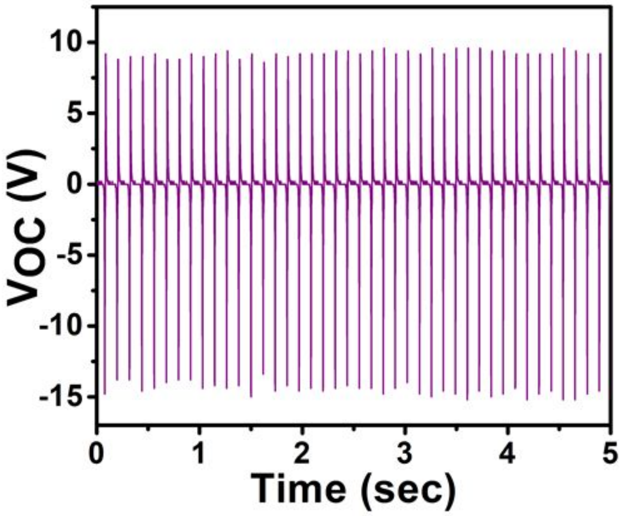

Figure S31. Durability data for 15 wt % **1**-TPU after a resting period of three months.

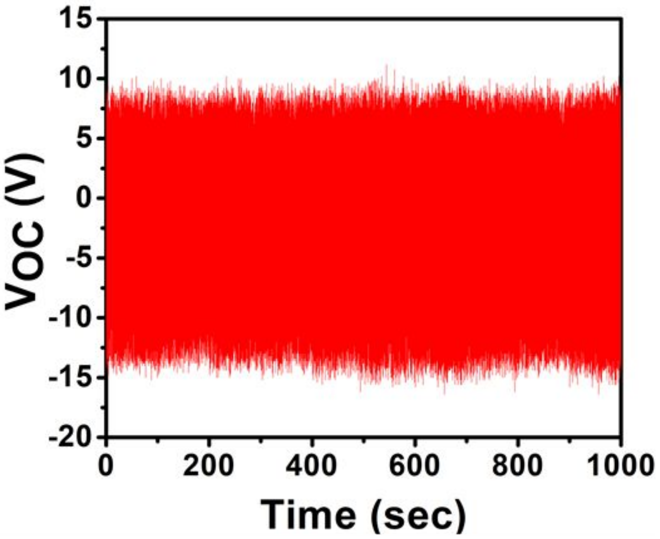

Figure S32. Fatigue test for the 15 wt % **1**-TPU composite device with a continuous applied force of 14.15 N over a period of 10000 cycles.

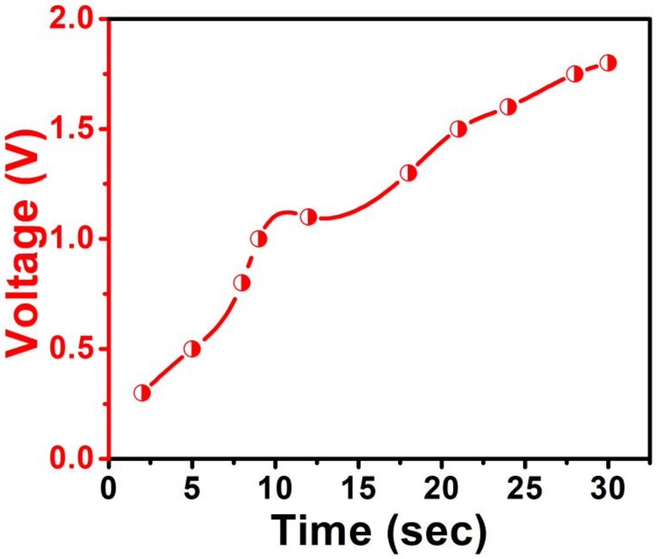

Figure S33. Stored voltages in a 100 μF capacitor by employing the 15 wt % **1**-TPU composite device at different time intervals.

References

1. Essawi, M. M. E. I., Synthesis and characterization of triphenylmethylphosphonium transition metal salts. *Transit. Met.Chem.* **1997,** *22*, 117-122.

2. Farra, R.; Thiel, K.; Winter, A.; Klamroth, T.; Pöppl, A.; Kelling, A.; Schilde, U.; Taubert, A.; Strauch, P., Tetrahalidocuprates(ii)—structure and EPR spectroscopy. Part 1:Tetrabromidocuprates(ii). *New J. Chem.* **2011,** *35*, 2793-2803.

3. Jella, V.; Ippili, S.; Eom, J.-H.; Choi, J.; Yoon, S.-G., Enhanced output performance of a flexible piezoelectric energy harvester based on stable MAPbI3-PVDF composite films. *Nano Energy* **2018,** *53*, 46-56.

4. Sultana, A.; Alam, M. M.; Sadhukhan, P.; Ghorai, U. K.; Das, S.; Middya, T. R.; Mandal, D., Organo-lead halide perovskite regulated green light emitting poly (vinylidene fluoride) electrospun nanofiber mat and its potential utility for ambient mechanical energy harvesting application. *Nano Energy* **2018,** *49*, 380-392.

5. Kim, Y.-J.; Dang, T.-V.; Choi, H.-J.; Park, B.-J.; Eom, J.-H.; Song, H.-A.; Seol, D.; Kim, Y.; Shin, S.-H.; Nah, J., Piezoelectric properties of CH 3 NH 3 PbI 3 perovskite thin films and their applications in piezoelectric generators. *J. Mater. Chem A* **2016,** *4*, 756-763.

6. Ding, R.; Liu, H.; Zhang, X.; Xiao, J.; Kishor, R.; Sun, H.; Zhu, B.; Chen, G.; Gao, F.; Feng, X., Flexible piezoelectric nanocomposite generators based on formamidinium lead halide perovskite nanoparticles. *Adv. Funct. Mater.* **2016,** *26*, 7708-7716.

7. Li, Y.; Xu, M.-h.; Xia, Y.-s.; Wu, J.-m.; Sun, X.-k.; Wang, S.; Hu, G.-h.; Xiong, C.-x., Multilayer assembly of electrospun/electrosprayed PVDF-based nanofibers and beads with enhanced piezoelectricity and high sensitivity. *Chem. Eng. Sci.* **2020,** *388*, 124205.

8. Tusiime, R.; Zabihi, F.; Tebyetekerwa, M.; Yousry, Y. M.; Wu, Y.; Eslamian, M.; Yang, S.; Ramakrishna, S.; Yu, M.; Zhang, H., High stress-driven voltages in net-like layer-supported organic–inorganic perovskites. *J. Mater. Chem. C* **2020,** *8*, 2643-2658.

9. Deswal, S.; Singh, S. K.; Rambabu, P.; Kulkarni, P.; Vaitheeswaran, G.; Praveenkumar, B.; Ogale, S.; Boomishankar, R., Flexible Composite Energy Harvesters from Ferroelectric A2MX4-Type Hybrid Halogenometallates. *Chem. Mater.* **2019,** *31*, 4545-4552.

10. Zhang, Y.; Song, X.-J.; Zhang, Z.-X.; Fu, D.-W.; Xiong, R.-G., Piezoelectric Energy Harvesting Based on Multiaxial Ferroelectrics by Precise Molecular Design. *Matter* **2020,** *2*, 697-710.

11. Vijayakanth, T.; Ram, F.; Praveenkumar, B.; Shanmuganathan, K.; Boomishankar, R., All-Organic Composites of Ferro-and Piezoelectric Phosphonium Salts for Mechanical Energy Harvesting Application. *Chem. Mater.* **2019,** *31*, 5964-5972.

12. Zhang, Y.; Hopkins, M. A.; Liptrot, D. J.; Khanbareh, H.; Groen, P.; Zhou, X.; Zhang, D.; Bao, Y.; Zhou, K.; Bowen, C. R., Harnessing Plasticity in an Amine-Borane as a Piezoelectric and Pyroelectric Flexible Film. *Angew. Chem. Int. Ed.* **2020,** *59*, 7808-7812.

13. Vijayakanth, T.; Ram, F.; Praveenkumar, B.; Shanmuganathan, K.; Boomishankar, R., Piezoelectric Energy Harvesting from a Ferroelectric Hybrid Salt [Ph3MeP] 4 [Ni (NCS) 6] Embedded in a Polymer Matrix. *Angew. Chem. Int. Ed.* **2020,** *59*, 10368-10373.
